# Exploring the relationship between sarcopenia and 11 respiratory diseases: a comprehensive mendelian randomization analysis

**DOI:** 10.1007/s40520-024-02855-y

**Published:** 2024-10-12

**Authors:** Yue Su, Youqian Zhang, Di Zhang, Jinfu Xu

**Affiliations:** 1 Department of Respiratory and Critical Care Medicine, School of Medicine, Shanghai Pulmonary Hospital, Tongji University, No. 507 Zhengmin Road, Shanghai, 200433 China; 2 Institute of Respiratory Medicine, School of Medicine, Tongji University, Shanghai, China; 3 Health Science Center, Yangtze University, Hubei Province, Jingzhou, 434000 China; 4 Chengdu University of Traditional Chinese Medicine, Sichuan Province, Chengdu, 610075 China

**Keywords:** Sarcopenia, Respiratory diseases, Mendelian randomization, Nutrition

## Abstract

**Background:**

Sarcopenia (SP) is an aging-related loss of muscle mass and function, affecting the respiratory system. However, the causality of the association between sarcopenia on lung diseases remains elusive.

**Methods:**

The bidirectional univariate Mendelian randomization (UVMR), multivariate MR (MVMR) analysis, and mediation MR were utilized to systematically investigate the genetic causal relationship of SP and 11 respiratory diseases. Independent genomic variants related to sarcopenia or respiratory diseases were identified as instrumental variables (IVs), and the summary level data of genome-wide associated studies (GWAS) were obtained from the UK biobank and FinnGen. MVMR analysis was conducted to explore the mediation effects of body mass index (BMI), Alcohol Use Disorders Identification Test (AUDIT), smoking, education attainment (EA), physical activity, and Type 2 Diabetes Mellitus (T2DM).

**Results:**

Forward UVMR analysis based on the primary method revealed that pneumoconiosis was associated with a higher risk of appendicular lean mass (ALM) (OR = 1.01, *p* = 0.03), and BMI (10.65%), smoking (10.65%), and physical activity (17.70%) had a mediating role in the effect of pneumoconiosis on ALM. In reverse MR analysis, we found that genetically predicted ALM was significantly associated with an increased risk of pulmonary embolism (PE) (OR = 1.24, *p* = 7.21E-05). Chronic obstructive pulmonary disease (COPD) (OR = 0.98, *p* = 0.002) and sarcoidosis (OR = 1.01, *p* = 0.004) were identified to increase the loss of left-hand grip strength (HGS). Conversely, the increase in left- HGS presented a protective effect on chronic bronchitis (CB) (OR = 0.35, *p* = 0.03), (OR = 0.80, *p* = 0.02), and asthma (OR = 0.78, *p* = 0.04). Similarly, the loss of the right-HGS elevated the risk of low respiratory tract infection (LRTI) (OR = 0.97, *p* = 0.02) and bronchiectasis (OR = 1.01, *p* = 0.03), which is also an independent protective factor for LRTI and asthma. In the aspects of low HGS, the risk of LRTI was increased after MVMR analysis, and the risk of sarcoidosis and pneumoconiosis was elevated in the reverse analysis. Lastly, asthma was found to be related to the loss of the usual walking pace, and the reverse MR analysis suggested a causal relationship between the usual walking pace and LRTI (OR = 0.32, *p* = 2.79 × 10−^5^), asthma (OR = 0.24, *p* = 2.09 × 10−^6^), COPD (OR = 0.22, *p* = 6.64 × 10−^4^), and PE(OR = 0.35, *p* = 0.03).

**Conclusions:**

This data-driven MR analysis revealed SP was bidirectional causally associated with lung diseases, providing genetic evidence for further mechanistic and clinical studies to understand the crosstalk between SP and lung diseases.

**Supplementary Information:**

The online version contains supplementary material available at 10.1007/s40520-024-02855-y.

## Introduction

Sarcopenia (SP) is a progressive and age-associated skeletal muscle disease characterized by reduced muscle mass and function that is related to increased rates of adverse events and hospitalization [1]. The consensus released by the European working group on sarcopenia in older people 2 and the US sarcopenia definition and outcome consortium suggests that reduced appendicular lean mass (ALM), left-hand grip strength (HGS), low HGS (60 years and older), and usual working pace are the main parameters reflecting the severity of sarcopenia [2, 3]. Lung diseases are respiratory system disorders that affect the airways, alveoli, interstitium, blood vessels, or pleura. Based on the summary data of the Global Burden Disease Study 2019 [4], it has been demonstrated that more than 500 million people have chronic pulmonary diseases across the world [5]. Emerging evidence has demonstrated that sarcopenia is associated with chronic obstructive pulmonary disease (COPD), and the patients with sarcopenia presented poorer prognosis [6, 7], however, the real clinical impact of sarcopenia on COPD remains elusive. Leem and colleagues have demonstrated that sarcopenia is related to cardiovascular risk in patients with COPD [8, 9]. Limpawattana et al. have shown that the prevalence of SP was approximately 25% in the enrolled COPD patients using the Asian Working Group for SP (AWGS) criteria in southeast Asia. Moreover, Minrata and colleagues have suggested that patients with bronchiectasis presented decreased muscle thickness and pennation angle [10]. Petermann-Rocha et al. have shown that pulmonary embolism (PE) patients with in-hospital mortality had a higher PE severity index and a lower value of psoas muscle, which was considered by the outcome of patients [11]. To date, most of the studies about SP with respiratory diseases focus on COPD.

Mendelian randomization (MR) is an analytic method of utilizing genetic variants as instrumental variables (IVs) to interrogate the causal impact of modifiable traits (exposure) on outcomes, possessing the ability to circumvent the confounding bias of observational research [12, 13]. Park and coworkers have suggested that tobacco smoking status contributes to poor aging phenotypes, and reducing tobacco use may result in decreasing the burden of SP associated-aging and frailty [14]. Similarly, Liu et al. have explored the association between COVID-19 and SP-related traits, highlighting elderly population with good nutrition status is capable of coping with SP during the COVID pandemic. However, the role of SP on common respiratory disorders and the impact of lung diseases on SP also need further study. This study aimed to reveal the causal relationship between respiratory diseases and SP-related traits by performing MR analysis. Furthermore, multivariable MR (MVMR) and mediation MR analyses were conducted to assess the mediating effects of six confounding factors.

## Methods

### Study design

**Fig. 1:**
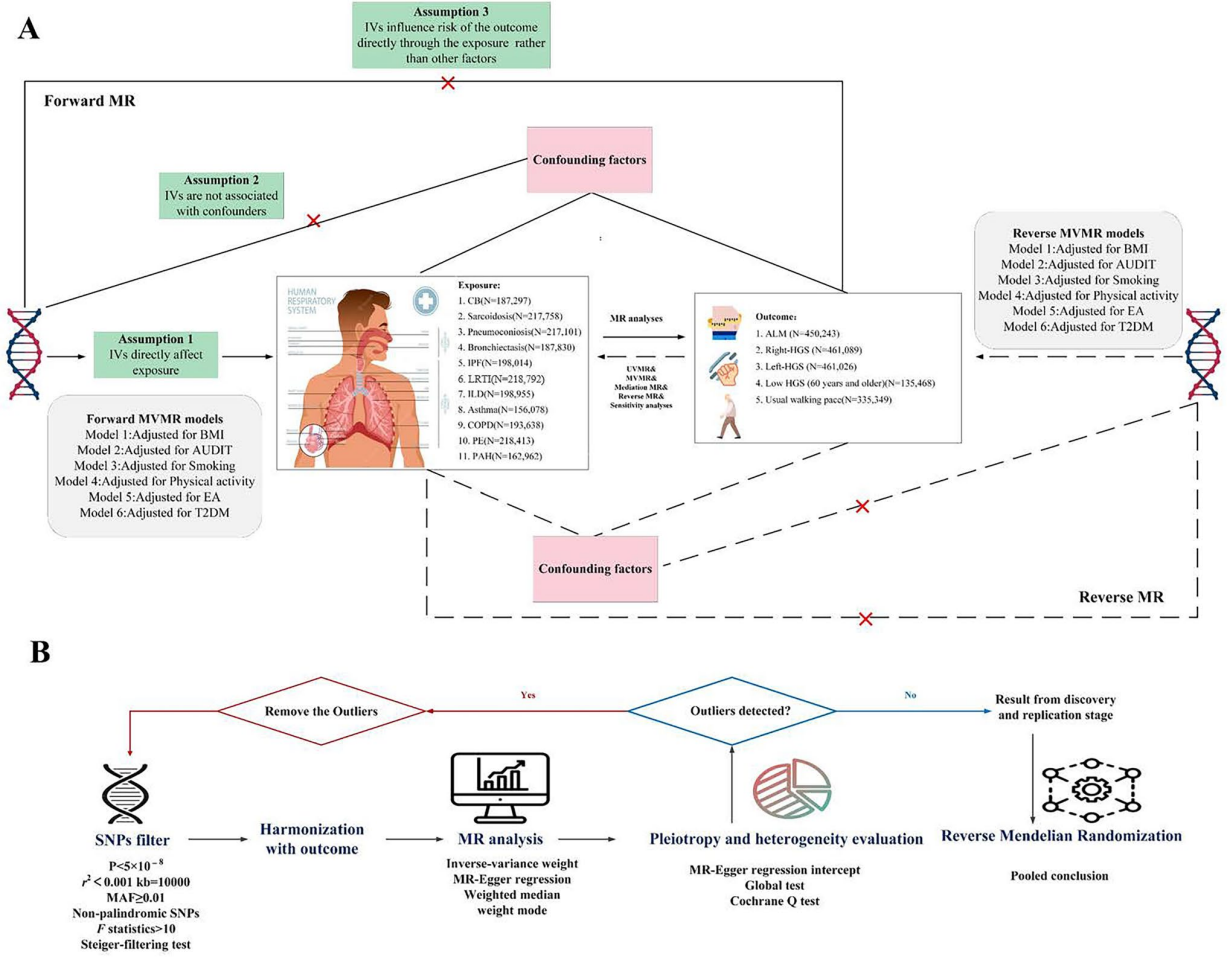
Overview of research design and analysis strategy. **A**: Overview of the research design. Exposures come from 11 respiratory diseases, with outcomes including five traits of sarcopenia. The MR framework is based on the those fundamental MR assumptions, with MVMR analyses adjusting for six mediating factors for all positive results. **B**: Analysis stratagy of MR. Eligible SNPs ars screened as IVs followed by sensitivity analysis, as well as evaluations for heterogeneity and pleiotropy Further reverse analyses are conducted. *CB* Chronic bronchitis, *IPF* Idiopathic pulmonary fibrosis, *ILD* Interstitial lung disania, *LRTI* Lower respiratory tract infection, *PAH* Pulmonary arterial hypertension, *PE* Pulmonary embolism, *COPD* Chronic obstructive pulmonary disease, *ALM* appendicular lean mass, *HGS* Hand grip strength, *MR* Mendelian randomization, *UVMR* Univariate MK, *BMI* Body mass index, *AUDIT* Alcohol use Disorders identification test, *EA* Education attainment, *T2DM* Type 2 diabetes mellitus

To investigate the potential causal relationships between 11 respiratory diseases (chronic bronchitis (CB), sarcoidosis, pneumoconiosis, bronchiectasis, idiopathic pulmonary fibrosis (IPF), LRTI, interstitial lung disease (ILD), asthma, COPD, PE, and pulmonary arterial hypertension (PAH)) and the risk of SP (low hand-grip strength, usual walking pace, and ALM), bidirectional univariate MR (UVMR) and MVMR analysis was developed. The choice of IVs for exposure hinged on three crucial assumptions: (i) the selected genetic variant, acting as the instrumental variable, demonstrates a robust association with the exposure; (ii) the genetic variant maintains no connection with potential confounders; and (iii) the influence of genetic variants on the outcome is mediated exclusively via the exposure, eliminating the possibility of alternate pathways [15]. Conversely, we probed the reciprocal impact of SP on 11 respiratory diseases, acknowledging the potential for reverse causation. Consequently, only single-nucleotide polymorphism (SNPs) demonstrating a genome-wide significance with outcome phenotype were considered to ensure a robust correlation between all instrumental variables and the exposure. Furthermore, these genetic variants needed to display independence and avoid linkage disequilibrium, signifying their random allocation at conception. In addition, as body mass index (BMI), Alcohol Use Disorders Identification Test (AUDIT), smoking, education attainment (EA), physical activity, and Type 2 Diabetes Mellitus (T2DM) may play confounding roles in the exposure to outcome pathway, further bidirectional MVMR analyses were conducted to estimate the direct causal effect of exposure on outcome. Compared with the UVMR hypothesis, assumption 1 of the MVMR refers to genetic variation linked with one or more exposures, and the remaining assumptions are aligned with the UVMR [16]. MVMR analysis was applied to assess whether the association of exposure with outcome could be affected by potential confounders, including BMI, EA, T2MD, smoking, AUDIT, and physical activity [16]. In addition, we also explored the proportion of mediating factors explaining. Initially, MR effect estimates were derived for each respiratory disorder about SP phenotypes utilizing the IVW method. Subsequently, multivariate MR analysis was implemented to quantify the impact of six mediators on the outcome while accounting for exposure factors. The product of these two estimates for each outcome was utilized to determine the indirect effect of exposure. Lastly, the mediators' contribution to the total effect was ascertained by dividing the mediating effect by the overall effect. The details of the MR design are displayed in Fig. 1

### Data sources

Genetic associations with 11 respiratory diseases were obtained from the FinnGen consortium. Instituted in Finland in 2017, the FinnGen study embarked on an expedition to collate and scrutinize genomic and health-related data from an expansive cohort of approximately half a million Finnish individuals (https://www.finngen.fi/en;).Summary-level statistics for right-HGS (461,089 individuals), left-HGS (461,026 individuals), and usual walking pace (335,349 individuals) were derived from the UK Biobank (UKB) for European ancestry participants. This UK-focused cohort study collected medical and physical data from roughly 500,000 participants, between 40 and 69 years old, from 2006 to 2010 [17]. GWAS summary statistics for ALM (450,243 individuals) and low HGS (60 years and older) (34,589 cases and 100,879 controls) were obtained from https://www.ebi.ac.uk/gwas/, a website maintained by the European Bioinformatics Institute (EBI) dedicated to storing and sharing data for GWAS. The EBI is Europe's largest bioinformatics resource provider [18].

Furthermore, we obtained genetic associations for BMI from the Genetic Investigation of Anthropometric Traits (GIANT) consortium [19], alcohol use from the Psychiatric Genomics Consortium (PGC) [20], T2DM from the DIAbetes Genetics Replication And Meta-analysis (DIAGRAM) consortium [21], EA from the GWAS of 1.1 million individuals conducted by the Social Science Genetic Association Consortium [22], physical activity from the family GWAS consortium [23] and smoking were identified from the GWAS and Sequencing Consortium of Alcohol and Nicotine use, involving 1.2 million individuals [24].

Each investigation integrated within the GWAS framework received approval from relevant ethical review panels. Written informed consent was obtained from all participants involved. The data used in this study remain publicly accessible. Summary information can be found in Table 1.

**Table 1:** Detailed information of data sources

Explore or Outcome	Ref	Ancestry	Participants	Web Source
Appendicular lean mass	33,097,823	European	450,243 individuals	https://gwas.mrcieu.ac.uk/datasets/ebi-a-GCST90000025/
Hand grip strength(right)	NA	European	461,089 individuals	https://gwas.mrcieu.ac.uk/datasets/ukb-b-10215/
Hand grip strength(left)	NA	European	461,026 individuals	https://gwas.mrcieu.ac.uk/datasets/ukb-b-7478/
Low hand grip strength(60 years and older)(EWSOP)	33,510,174	European	34,589 individuals	https://gwas.mrcieu.ac.uk/datasets/ukb-b-7478/
Usual walking pace	NA	European	335349individuals	https://gwas.mrcieu.ac.uk/datasets/ukb-a-513/
chronic bronchitis	NA	European	574 cases and 186,723 controls	https://gwas.mrcieu.ac.uk/datasets/finn-b-J10_SIMPLBRONCH/
Sarcoidosis	NA	European	2046 cases and 215,712 controls	https://gwas.mrcieu.ac.uk/datasets/finn-b-D3_SARCOIDOSIS/
Pneumoconiosis	NA	European	235 cases and 216,866 controls	https://gwas.mrcieu.ac.uk/datasets/finn-b-J10_ASBESTPNEUMOC/
Bronchiectasis	NA	European	1107 cases and 186,723 controls	https://gwas.mrcieu.ac.uk/datasets/finn-b-J10_BRONCHIECTASIS/
idiopathic pulmonary fibrosis	NA	European	1028 cases and 196,986 controls	https://gwas.mrcieu.ac.uk/datasets/finn-b-IPF/
lower respiratory tract infection	NA	European	32,069 cases and 186,723 controls	https://gwas.mrcieu.ac.uk/datasets/finn-b-J10_LOWCHRON/
Interstitial lung disease	NA	European	1969 cases and 196,986 controls	https://gwas.mrcieu.ac.uk/datasets/finn-b-ILD/
Asthma	NA	European	20,629 cases and 135,449 controls	https://gwas.mrcieu.ac.uk/datasets/finn-b-J10_ASTHMA/
COPD	NA	European	6915 cases and 186,723 controls	https://gwas.mrcieu.ac.uk/datasets/finn-b-J10_COPD/
pulmonary embolism	NA	European	4185 cases and 214,228 controls	https://gwas.mrcieu.ac.uk/datasets/finn-b-I9_PULMEMB/
PAH	NA	European	125 cases and 162,837 controls	https://gwas.mrcieu.ac.uk/datasets/finn-b-I9_HYPTENSPUL/
Physical activity	NA	European	78,077 individuals	https://gwas.mrcieu.ac.uk/datasets/ieu-b-4860/
BMI	30,239,722	European	694,649 individuals	https://pubmed.ncbi.nlm.nih.gov/30239722/
AUDIT	30,336,701	European	141,932 individuals	https://www.ncbi.nlm.nih.gov/pmc/articles/PMC6365681/
T2DM	30,297,969	European	74,124 cases and 824,006 controls	https://pubmed.ncbi.nlm.nih.gov/30297969/
EA	30,038,396	European	1,100,000 individuals	https://pubmed.ncbi.nlm.nih.gov/30038396/
smoking	30,643,251	European	1,200,000 individuals	https://pubmed.ncbi.nlm.nih.gov/30643251/

*BMI* Body mass index, *T2DM* Type 2 diabetes mellitus, *EA* Education attainment, *PAH* Pulmonary arterial hypertension, *COPD* Chronic obstructive pulmonary disease

Selection of genetic instrumental variables.

Within the framework of our MR studies, we treated the included SNPs as genetic IVs. To ensure the accuracy of the MR estimates, these SNPs were required to satisfy specific criteria:

(1) All SNPs chosen as IVs manifested a correlation with the respective exposure at a genome-wide significance threshold (*P* < 5 × 10^−8^). Since no genome-wide significant SNPs for COPD and Pneumoconiosis, we adopted a less stringent threshold of 5 × 10^−6^ to obtain more SNPs for these phenotypes [25].

(2) SNPs were ensured to be unassociated with any potential confounders and independent of one another to prevent biases stemming from linkage disequilibrium (r^2^ < 0.001, clumping distance = 10,000 kb) [26];

(3) F-statistics were employed to test for weak instrumental variables, and all of the F-statistics of the incorporated SNPs exceeded 10. A larger F-statistic indicates stronger instrument strength. We calculated an F-statistic (F = beta^2^/se^2^; beta for the SNP-exposure association (beta); variance (se)) for each SNP [27].

(4) To maintain the robustness of the results, MR-Steiger filtering was employed to eliminate variations demonstrating stronger correlations with outcomes than with exposures [28].

### Statistical analysis

The R programming language (version 4.2.3) and the R packages "TwoSampleMR" "MRPRESSO" and "MendelR" were used to conduct all statistical and MR studies. Initially, the multiplicative random-effects IVW method served as the primary statistical model, enabling the estimation of the causal effects of 11 respiratory diseases on SP risk [29]. The main analysis was performed using the random effects IVW technique, which offers reliable and efficient estimates when all genetic variants are considered valid [30]. IVW weighting is connected to the precision estimation of the Wald ratio for each SNP and is inversely related to each SNP's Wald ratio variance estimation. The weighted median method performs better when half of the genetic variants are deemed invalid [31]. Additional validation of MR estimates was achieved through supplementary analyses utilizing four separate methods. Considering the Bonferroni correction for multiple tests, a *p-value* less than 0.01/0.005 (Forward:0.05/5 Reverse: 0.05/11) is considered statistically significant evidence of a causal association. A *p-value* of less than 0.05 is considered suggestive evidence for a potential causal association.

To ensure reliable MR estimates, a series of sensitivity analyses were conducted. Cochran’s Q test was utilized to evaluate the heterogeneity of each genetic variant, with a P-value under 0.05 indicating significant heterogeneity among the selected SNPs [32]. Directional pleiotropy in our MR study was scrutinized using MR-Egger regression [33], and a P-value below the 0.05 threshold concerning the MR-Egger's intercept may suggest significant directional pleiotropy. Although the MR-Egger method has relatively lower accuracy, its intercept can indicate the presence of directional pleiotropy [34]. The MR-PRESSO method was employed to identify potential outliers and investigate horizontal pleiotropy, which is presumed if the global P-value falls below 0.05 [35]. Any outliers would be removed to enhance the accuracy of the correction. Lastly, a leave-one-out analysis was conducted to evaluate the impact of individual SNPs on the overall results [36].

The effect of an SNP on 11 respiratory diseases and the effect of that SNP on SP must each correspond to the same allele. This SNP will be excluded if the alleles do not correspond to the same SNP. If an SNP is absent in the outcome dataset, we employ the SNiPa online tool (http://snipa.helmholtz-muenchen.de/snipa3/) to locate the respective SNP. This tool utilizes European population genotype data derived from Phase 3 of the 1000 Genomes Project. Subsequently, another SNP exhibiting linkage disequilibrium (r^2^ > 0.8) with the initial SNP is identified as a proxy SNP.

## Results

### Discovery results of SP

**Fig. 2:**
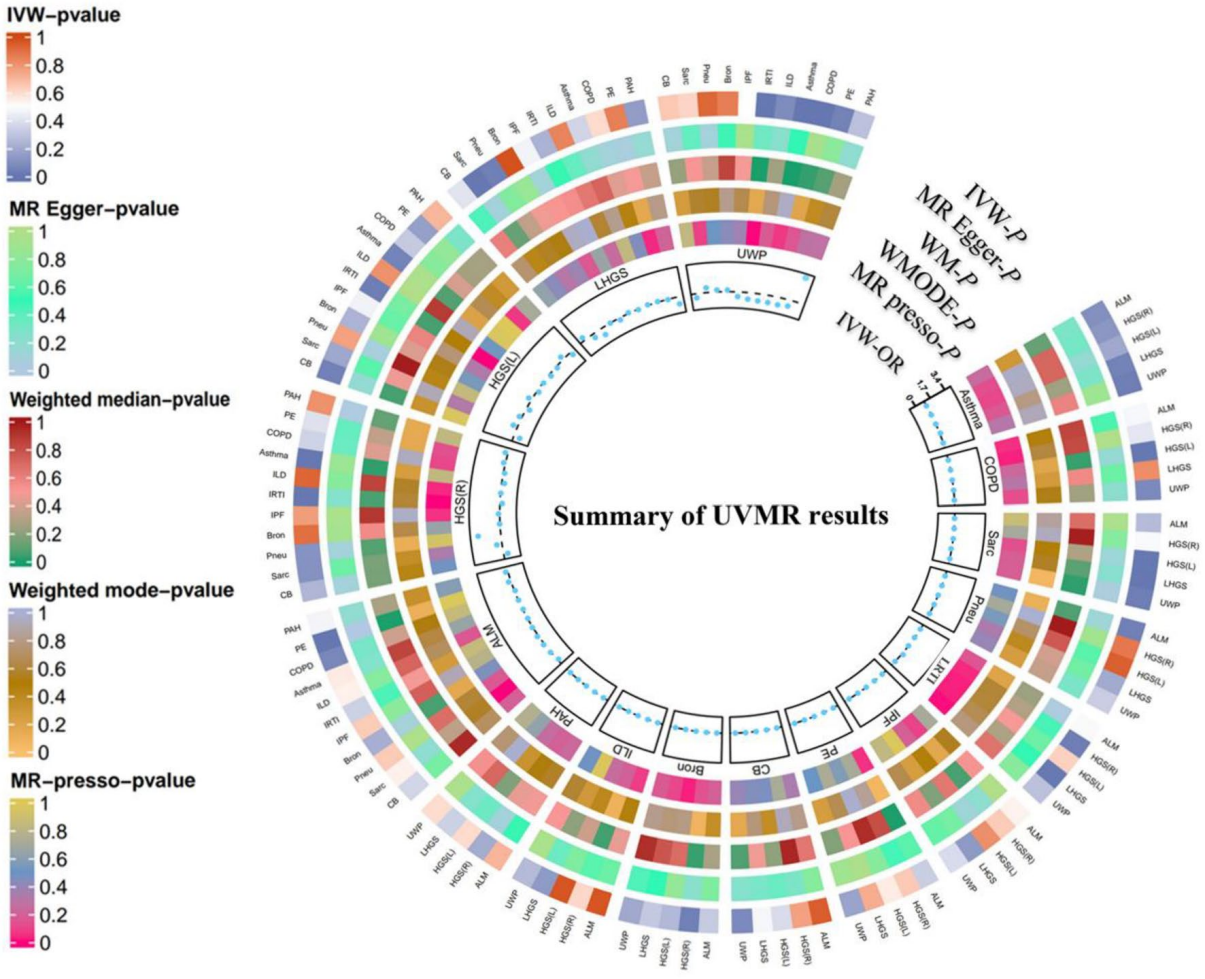
Bi-directional associations between sarcopenia characteristics and 11 respiratory diseases. *ALM* Appendicular lean mass *HGS(L)* Hand grip strength (left) *HGS(R)* Hand grip strength (right), *LHGS* Low hand grip strength, *UWP* Usual walking pace, *CB* Chronic bronchitis, *Sare* sarcoidosis, *Phau* Pneumoconiosis, *Bron* Bronchiectasis, *IPF* Idiopathic pulmonary fibrosis, *ILD* Interstitial lung disease, *LRTI* Lower respiratory tract infection, *PAH* Pulmonary arterial hypertension, *PE* pulmonary embolism, *COPD* Chronic obstructive pulmonary disease, *UVMR* Univariate mendelian randomization, *IVW* Inverse variance weighting, *WM* Weighted median, *WMODE* Weighted mode, *MR.presso*: MR Pleiotropy residual sum and outlier, *OR* Odds ratio

**Fig. 3:**
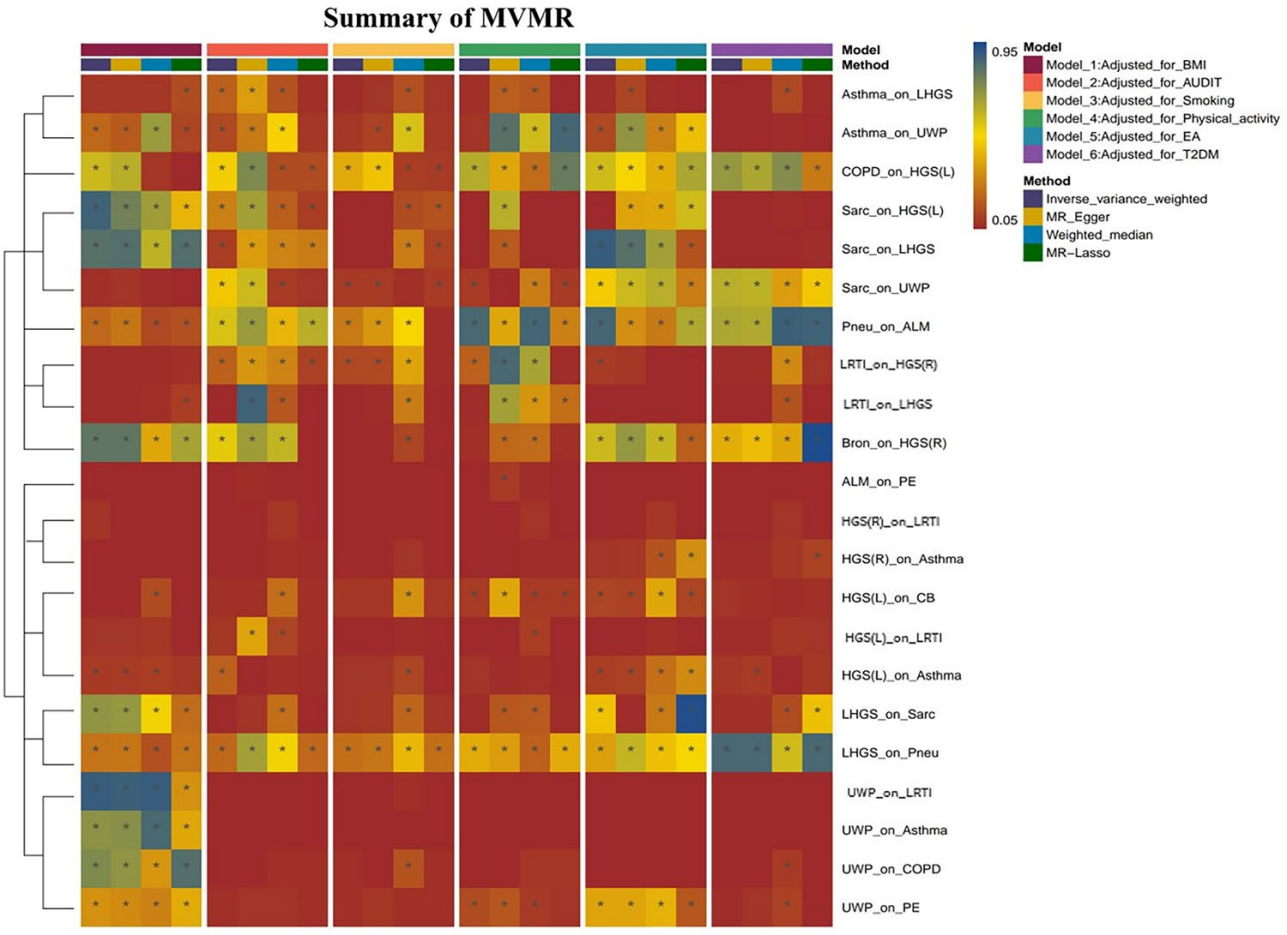
MVMR analysis of positive associations between sarcopania traits and 11 respiratory diseases. The graph shows all positive rostules in both forward and reverse analyses after adjusting for six confounding factors, with P-values displayed using four different methods. *MVMR* multivariats Mendelian randomization, *BMI* Body mass index, *AUDIT* Alcohol use Disorders identification test, *EA* Education attainment, *T2DM* Type 2 diabetes mellitus, *ALM* Appendicular lean mass, *HGS(L)* Hand grip strangth (laft), *HGS(R)* Hand grip strength (right), *LHGS* Low hand grip strength, *UWP* usual walking pace, *COPD* Chronic obstructive pulmonary disease, *Sare* sarcoidosis, *Preu*, *LKTI* Lower respiratory tract infection, *Bron* Bronchiectasis, *PE* Pulmonary embolism, *CB* Chronic bronchitis, *: Indicates loss of causality due to confounding factors, p > 0.05.

**Fig. 4:**
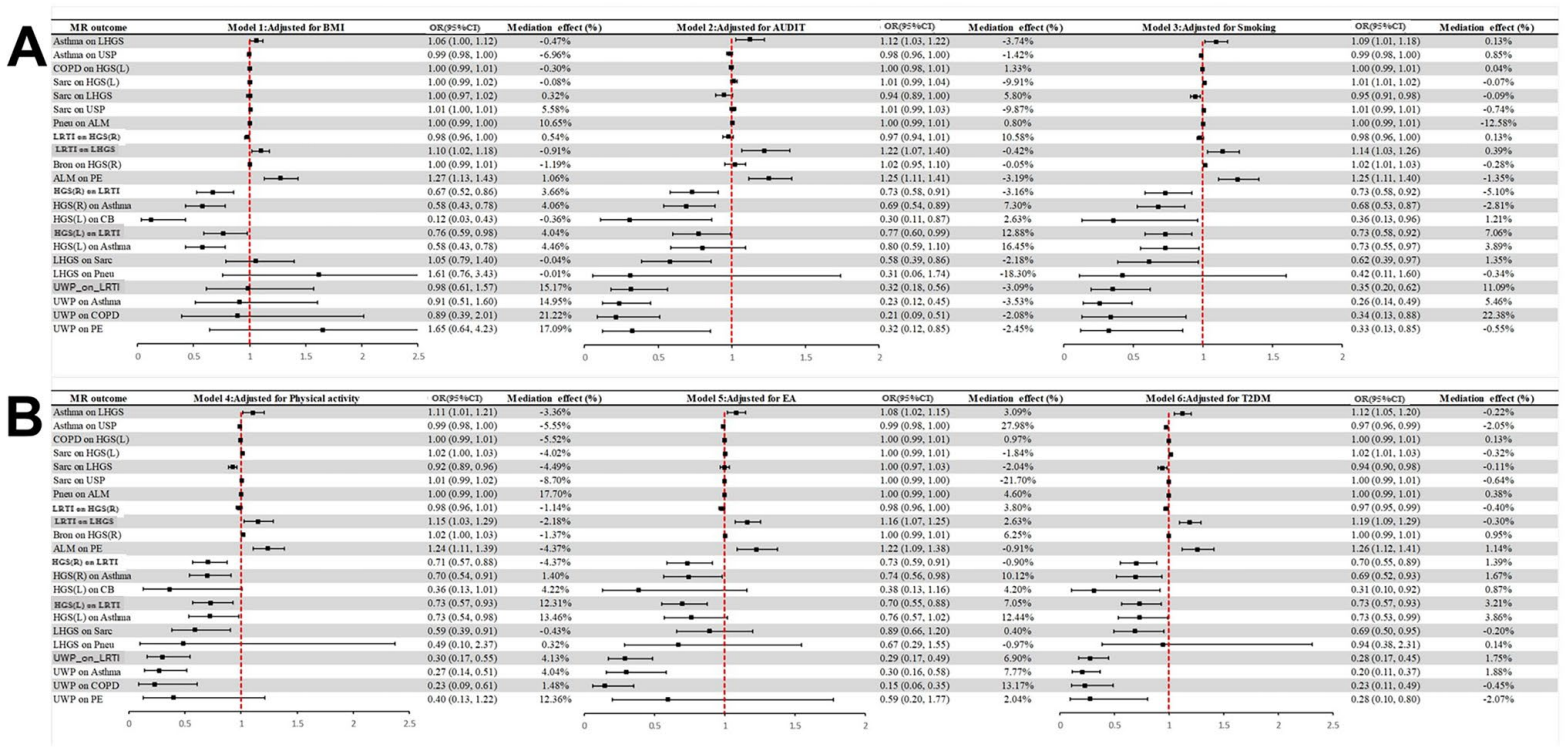
Estimating the impact of all positive exposure factors on the outcome through potential mediators. **A** represents the causal effects of all exposures after receiving adjustments for BMI, AUDIT, and smoking. **B**: shows the causal effects of all exposures after adjustment for PHYSICAL ACTIVITY, EA, and T2DM. *BMI* Body mass index, *AUDIT* Alcohol use disorders identification test, *EA* Education attainment, *T2DM* Type 2 diabetes mellitus, *ALM* Appendicular lean mass, *HGS(L)* Hand grip strength (laft), *HGS(R)* Hand grip strength (right), *LHGS* Low hand grip strength, *UWP* Usual walking pace, *COPD* Chronic obstructive pulmonary disease, *Sarc* Sarcoidosis, *Pneu* Pneumoconiosis, *LRTI* Lower respiratory tract infection, *Bron* Bronchiectasis, *PE* Pulmonary embolism, *CB* Chronic bronchitis, *OR* Odds ratio, *CI* Confidence interval

The discovery results of SP are summarized in Fig. 2 and forward and reverse are detailed in the subsequent sections (Table. S1–S2). All sensitivity results are summarised in Table S3-S4. The full results of the MVMR analysis are shown in Fig. 3. The full results of mediator MR are shown in Fig. 4. Detailed results are given in Table S5. All forest plots, leave-one-out plots, scatter plots, and funnel plots for positive results are shown in Fig. S1–S22.

### Risk factors for ALM

The IVW results indicated a potential causal effect of pneumoconiosis on ALM after Bonferroni correction, with genetically predicted pneumoconiosis raising the risk of ALM (OR = 1.002, 95%CI (1.001, 1.004), *p* = 0.033). However, after adjusting for confounders of MVMR, the risk was not statistically significant. We noted that BMI (10.65%), smoking (10.65%), and physical activity (17.70%) played a considerable mediating role in the effect of pneumoconiosis on ALM.

In reverse MR analysis, we found that genetically predicted ALM was significantly associated with an increased risk of PE (OR = 1.239, 95%CI (1.115, 1.378), *p* = 7.21E-05). Further MVMR analysis suggested that ALM was an independent risk factor for PE. However, no causal association was noted between other respiratory diseases and ALM. (all *p* > 0.05).

### Risk factors for left-HGS

Among left-HGS, we found two significant causal relationships after correction, for COPD (OR = 0.989, 95%CI (0.983,0.996), *p* = 0.002), and sarcoidosis (OR = 1.009, 95%CI (1.002,1.016), *p* = 0.004), respectively. However, the risk was not significant after adjusting for confounding factors in MVMR. The results for sarcoidosis remained significant after adjusting for smoking (*p* = 0.001), physical activity (*p* = 0.007), EA (*p* = 0.002), and T2MD (*p* = 0.003). In the reverse MR analysis, IVW identified three potential protective causal relationships by correction, namely CB (OR = 0.357, 95%CI (0.136, 0.937), *p* = 0.036), LRTI (OR = 0.802, 95%CI (0.659, 0.976), *p* = 0.028), and asthma (OR = 0.781, 95%CI (0.612, 0.997), *p* = 0.047).

Further MVMR states that left-HGS is an independent protective factor for LRTI. CB adjusted for physical activity (*p* = 0.003), EA (*p* = 0.003), and similarly, Asthma was no longer significant after adjusting for AUDIT and EA.

### Risk factors for right-HGS

Our study identified two significant causal relationships associated with right-HGS, specifically LRTI (OR = 0.979, 95%CI (0.961, 0.998), *p* = 0.029), and bronchiectasis (OR = 1.005, 95%CI (1.001, 1.010), *p* = 0.033). Nonetheless, the significance of LRTI and bronchiectasis decreased after MVMR adjustments for AUDIT score (*p* = 0.16), smoking (*p* = 0.09), physical activity (*p* = 0.16), and EA (*p* = 0.06), and all confounders except for physical activity, respectively. Mediation effects were 10.58%, 0.13%, −1.14%, and 3.80% for LRTI, and similar for bronchiectasis. In the reverse MR analysis using IVW, we found two significant protective causal relationships for LRTI (OR = 0.772, 95%CI (0.642, 0.927), *p* = 0.005) and asthma (OR = 0.734, 95%CI (0.589, 0.914), *p* = 0.005). After MVMR adjustments, right-HGS remained an independent protective factor for both diseases. There was no causal association between other respiratory diseases and right- HGS (all *p* > 0.05).

### Risk factors for low HGS

In the case of low HGS, our findings showed two significant causal relationships: sarcoidosis (OR = 0.939, 95%CI (0.909, 0.971), *p* = 2.22 × 10^−4^) and LRTI (OR = 1.135, 95%CI (1.035, 1.246), *p* = 0.007). Additionally, a potential causal relationship was observed with asthma (OR = 1.073, 95%CI (1.003, 1.149), *p* = 0.040). However, following MVMR analysis, only LRTI emerged as the independent risk factor for low HGS. The associations with asthma and sarcoidosis became insignificant after adjusting for various confounders: AUDIT scores (*p* = 0.16, mediation effect = −3.74%), BMI (*p* = 0.86, mediation effect = 0.32%), AUDIT (*p* = 0.06, mediation effect = 5.80%), and EA (*p* = 0.93, mediation effect = −2.04%). In the reverse MR analysis, sarcoidosis and pneumoconiosis showed a significant and potential causal relationship respectively but lost their significance after adjusting for the confounders. No causal association was identified between other respiratory diseases and low HGS (all *p* > 0.05).

### Risk factors for usual walking pace

We found potential causal links between the usual walking pace with asthma (OR = 0.989, 95%CI (0.981, 0.999), *p* = 0.02) and sarcoidosis (OR = 1.006, 95%CI (1.001, 1.011), *p* = 0.03). However, these associations became non-significant when adjusted for BMI (*p* = 0.18, mediation effect = −6.96%) and AUDIT (*p* = 0.09, mediation effect = −1.42%) for asthma, and AUDIT (*p* = 0.45, mediation effect = -9.87%), smoking (*p* = 0.059, mediation effect = −0.74%), physical activity (*p* = 0.0502, mediation effect = −8.70%), and T2DM (*p* = 0.64, mediation effect = -0.64%) for sarcoidosis (all *p* > 0.05).

Reverse MR revealed causal relationships with LRTI (OR = 0.320, 95%CI(0.188, 0.546), *p* = 2.79 × 10^−5^), asthma (OR = 0.246, 95%CI(0.138, 0.439), *p* = 2.09 × 10^−6^), and COPD(OR = 0.221, 95%CI(0.093, 0.526), *p* = 6.64 × 10^−4^), and a potential causal with PE (OR = 0.353, 95%CI(0.131, 0.953), *p* = 0.03).Further MVMR analysis showed that all four associations were not significant after adjusting for BMI (*p* > 0.05), with mediation effect values of 15.17%, 14.95%, 21.22%, and 17.09%, respectively. Further adjustment for physical activity (*p* = 0.10, mediation effect = 12.36%) and EA (*p* = 0.35, mediation effect = 2.04%) also nullified PE's causal link. No other significant causal associations between respiratory diseases and usual walking pace were found (all *p* > 0.05).

## Discussion

We conducted an exhaustive MR study to discern the association between genetic predisposition for 11 distinct respiratory diseases and traits related to SP. We discovered that specific diseases demonstrated positive correlations with only certain traits in SP, and therefore couldn't be directly characterized as strongly associated. However, among all the conditions investigated, asthma, LRTI, and sarcoidosis presented a more substantial association with SP and thus were classified as having a high degree of association. COPD, PE, and Pneumoconiosis presented moderate association, whereas CB and Bronchiectasis had a relatively weaker association with SP. Furthermore, our MVMR and mediation analyses underscored the significant mediating role of BMI, AUDIT, smoking, physical activity, EA, and T2DM in the causal links between the identified risk factors and SP-related traits.

Recent research has identified asthma's association with SP, with evidence indicating a positive correlation between asthma and decreased grip strength and walking speed. A series of observational studies corroborate this association [37–39], and our research further bolsters this hypothesis with comprehensive evidence from a genetic standpoint. The persistent inflammatory condition inherent in asthma may precipitate muscle atrophy and weakness, which are fundamental characteristics of SP. Systemic inflammation could potentially instigate protein degradation and impede protein synthesis within muscle tissues, culminating in muscle atrophy [40]. Conversely, muscle strength and function enhancements could augment overall health and flexibility, consequently diminishing the likelihood of chronic disease manifestation, including asthma [41]. As per the MVMR outcomes, both forward and reverse MR results are influenced by BMI and AUDIT. Both obesity and alcohol consumption incite systemic inflammation [42], which could subsequently intensify asthma and SP. Furthermore, the chronic administration of corticosteroids, a common therapeutic approach in asthmatic patients, can contribute to myopathy or muscle weakness [43]. Thus, management strategies for asthma and SP should consider these interrelated factors.

The impact of LRTI on SP is multifaceted. Evidence points to a positive correlation between LRTI and decreased grip strength, while increased grip strength and walking speed are inversely associated with LRTI risk. This relationship may be attributable to not only inflammatory triggers but also heightened metabolic demands, diminished physical activity, and hypoxia. Severe LRTI often precipitates decreased physical activity due to symptoms such as dyspnea, fatigue, or general malaise [10]. This decrease contributes to the progression of SP, as routine muscle engagement is vital for preserving muscle mass and strength. Moreover, the body's immunological response to LRTI substantially escalates metabolic demands [44]. In conjunction with reduced nutritional intake, this causes the body to utilize muscle protein as an energy source, leading to SP. Lastly, severe LRTI can induce hypoxia. Hypoxia-Inducible Factors (HIFs), which are activated under hypoxic conditions, can trigger catabolic pathways, leading to muscle atrophy [45]. Hence, the relationship between LRTI and SP is multi-dimensional and influenced by a wide array of physiological factors. Adjustments for factors such as physical activity and smoking, as per the MVMR analysis, substantiate the aforementioned associations. Furthermore, EA findings suggest that individuals with a higher level of education are likely to possess a greater capacity for comprehending and implementing healthy behaviors [46]. These behaviors directly affect muscle health, emphasizing education’s role in maintaining overall well-being.

Contrary to established research, our findings suggest sarcoidosis might enhance muscle function. This proposition contradicts established observational research [47, 48], yet the intricate interactions of factors in sarcoidosis do not preclude such outcomes. Several hypotheses are presented herein: initially, sarcoidosis patients may foster compensatory mechanisms to bolster muscle function in response to disease. Additionally, sarcoidosis, a primarily T-cell and macrophage-driven autoimmune disease, elicits a spectrum of immune responses [49]. Cytokines integral to these reactions, like IL-6 and TNF-α, typically rise in chronic inflammatory diseases such as sarcoidosis, stimulating muscle protein degradation, retarding protein synthesis, and consequently invoking catabolic processes in muscle tissue [50]. Conversely, certain aspects of the immune response may stimulate anabolic or muscle-building activity. For instance, T cells and macrophages are instrumental in muscle repair and regeneration following injury [51]. Immune responses in sarcoidosis might trigger analogous regenerative pathways, thereby enhancing muscle strength and function. Furthermore, corticosteroids, recognized for their potent anti-inflammatory properties, may, when used long-term by sarcoidosis patients, induce muscle atrophy and weakness [43]. Nonetheless, acute or intermittent use could offset inflammation-induced muscle damage, providing a temporary boost to muscle strength and performance [52]. This effect could be attributed to their impact on electrolyte balance and neuromuscular transmission. Lastly, other therapeutic strategies for sarcoidosis, such as immunosuppressants (methotrexate and azathioprine, for example), could have implications for muscle function. While they could elicit muscle pain and weakness, they may concurrently confer potential benefits to muscle function through systemic inflammation reduction [53]. These potential mechanisms underscore the complexity of the nexus among sarcoidosis, its treatment, and muscle function. To fully comprehend these intricate relationships and their implications for sarcoidosis patients, further investigation is warranted.

Our MR analysis affirmed the associations between SP traits and COPD [54], PE [55], bronchiectasis [11], and CB [56], as established in previous studies. We also discovered a novel link with pneumoconiosis. Pneumoconiosis, often found in heavy labor industries, could lead to an initial increase in ALM but also potentially precipitate SP as the disease progresses. As delineated earlier, the multifaceted pathophysiology of SP encompasses diminished oxygen supply, physical inactivity, inflammation, corticosteroid-induced side effects, and nutritional influences. Of note, pneumoconiosis displayed a positive correlation with ALM. The etiology of pneumoconiosis—extended exposure to mineral dust, such as coal or silica, in work environments [57]—often arises in industries demanding strenuous physical labor, such as mining and sandblasting. This initiative could increase ALM. However, we could not confirm causal links between PAH [58], ILD [59], IPF [60], and SP traits, possibly due to inherent biases in observational studies. Therefore, future MR analyses should leverage broader GWAS summary data and augment their genetic toolsets to substantiate the conclusions proposed in this study.

Our study possesses several strengths. It stands as the inaugural MR investigation offering a comprehensive analysis of respiratory system diseases and SP, along with their associated traits. The use of robust statistical tools on two distinct GWAS datasets (e.g., F-statistic significantly exceeding 10) to curtail bias potentially induced by overlapping samples. Moreover, all SNPs deployed as IVs were sourced from European populations, minimizing the risk of population stratification bias and bolstering the credibility of bidirectional MR hypotheses. In addition, through multivariate MR analyses, we elucidated intermediary pathways, deepening our comprehension of underlying mechanisms and providing supportive evidence for preventative strategies.

However, there are seveal limitations in this study. Firstly, a prominent limitation is an inability to definitively discount horizontal pleiotropy's influence. Despite this, the stability of associations was affirmed through numerous sensitivity analyses. Secondly, to preclude bias from overlapping samples, the small case proportion for certain diseases in the selected FinnGen database, thus warranting future analyses with larger GWAS sample data. Thirdly, it has been demonstrated that the Finnish population is not representative of European populations given its particular genetic characteristics [61]. Using single summary statistics data from the FinnGen and the UKBiobank may be more accurate to analyse the association between sarcopenia and lung diseases. Fourthly, for Pneumoconiosis, the causal effect of pneumoconiosis on ALM is not maintained after sensitivity analyses. Moreover, we only use the data from FinnGen, and the data from UK biobank should be analysed for detecting the association between Pneumoconiosis and sarcopenia. The last but not the least, the study's scope was confined to European samples, hence the results should be judiciously interpreted and corroborated within other genetic lineages.

## Conclusion

In the present study, a bidirectional MR study reveals the bidirectional causal relationship between sarcopenia and 11 lung diseases, providing new insights into the biological mechanisms of sarcopenia-regulated developments of lung diseases. The MVMR to assess the association of sarcopenia with respiratory diseases could be affected by selected potential confounders. To facilitate the dissection of the role of sarcopenia on lung diseases, an integrative approach that uses multiple omics is urgently needed to understand sarcopenia signalling.

## Supplementary Information

Below is the link to the electronic supplementary material.

Supplementary file1 (PPTX 1880 KB)

Supplementary file2 (PPTX 4385 KB)

Supplementary file3 (XLSX 25 KB)

Supplementary file4 (XLSX 25 KB)

Supplementary file5 (XLSX 19 KB)

Supplementary file6 (XLSX 20 KB)

Supplementary file7 (XLSX 16 KB)

## Data Availability

The public datasets used in this study are available in IEU Open GWAS Project(https://gwas.mrcieu.ac.uk/), FinnGen(https://www.finngen.fi/en/access_results).
