# Supplementary material for: Exploring the relationship between sarcopenia and 11 respiratory diseases: a comprehensive mendelian randomization analysis
Source: Aging Clin Exp Res. 2024 Oct 12;36(1):205. doi: 10.1007/s40520-024-02855-y (PMC11470909; doi:10.1007/s40520-024-02855-y)
Supplement: Supplementary file 1 — Supplementary file1 (PPTX 1880 KB) [file 40520_2024_2855_MOESM1_ESM.pptx]

## Slide 1
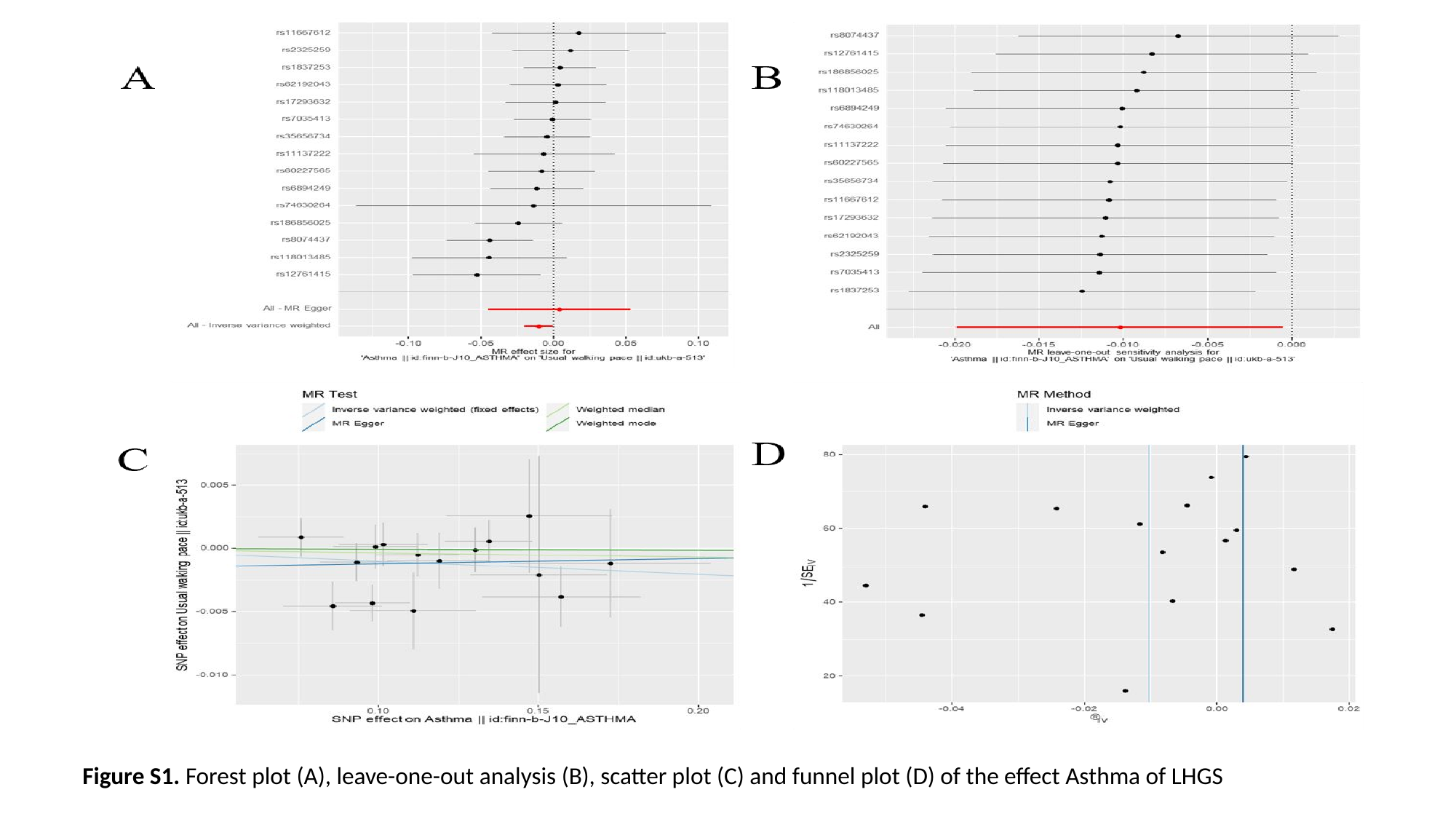

Figure S1. Forest plot (A), leave-one-out analysis (B), scatter plot (C) and funnel plot (D) of the effect Asthma of LHGS

## Slide 2
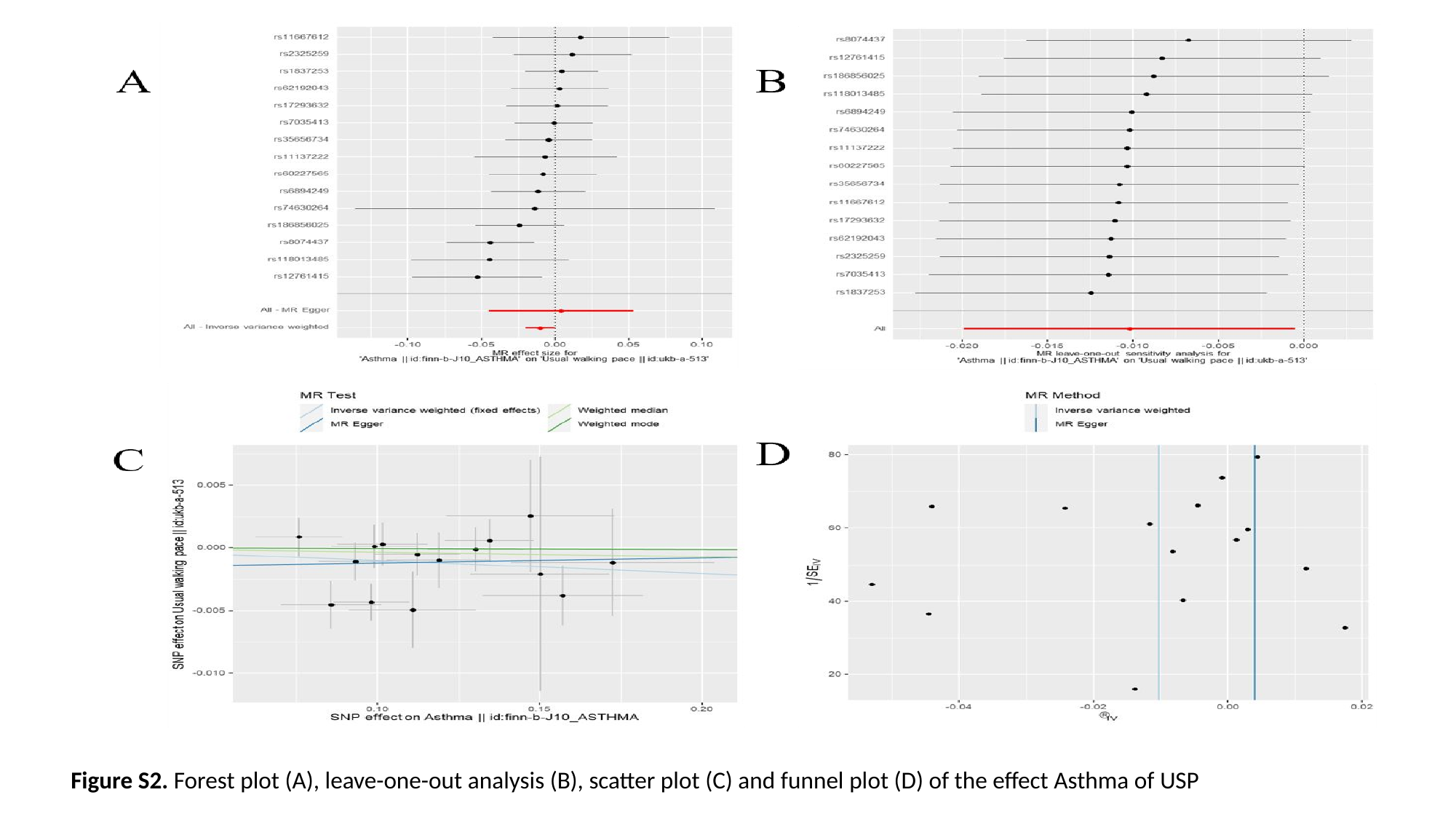

Figure S2. Forest plot (A), leave-one-out analysis (B), scatter plot (C) and funnel plot (D) of the effect Asthma of USP

## Slide 3
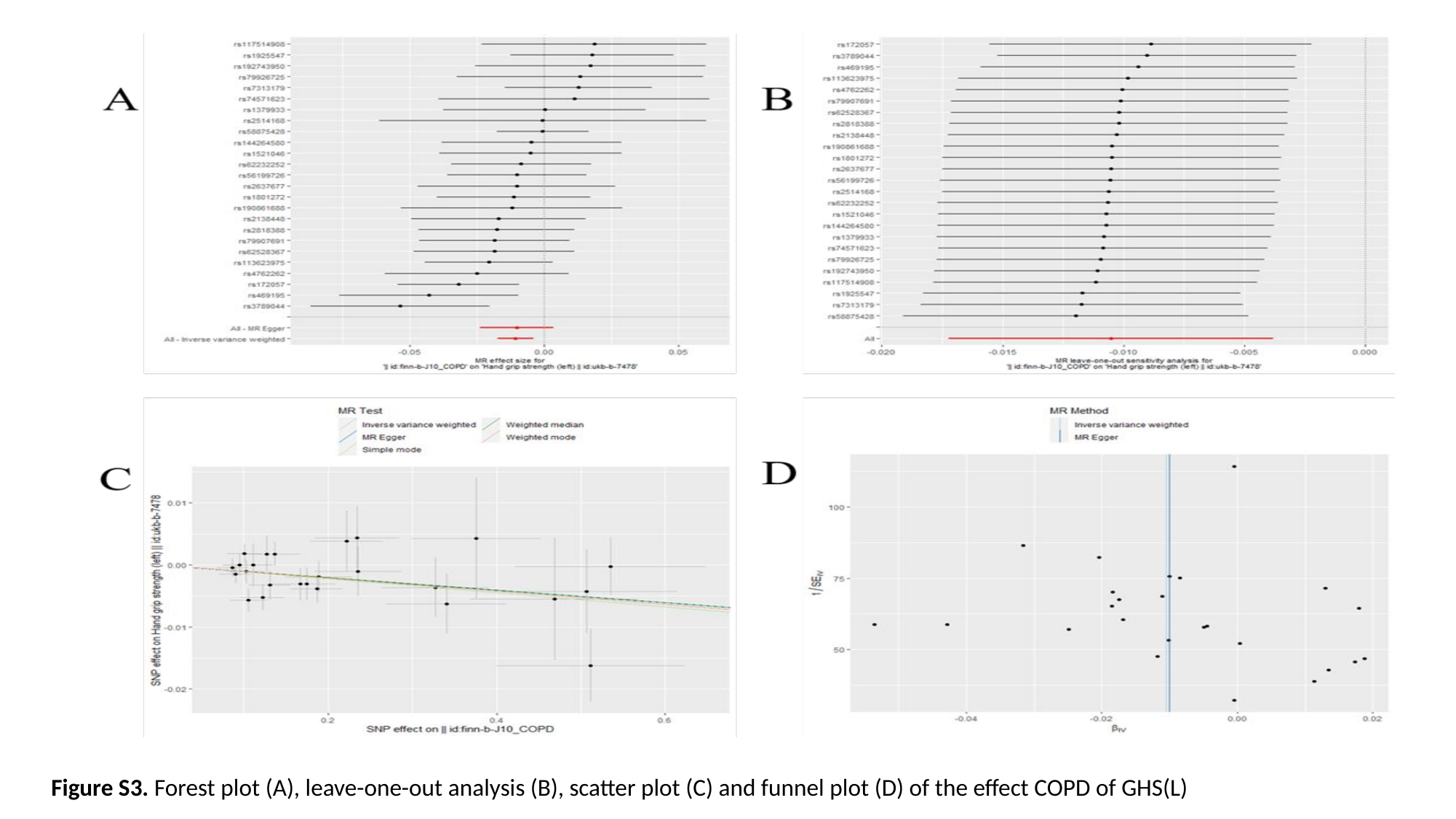

Figure S3. Forest plot (A), leave-one-out analysis (B), scatter plot (C) and funnel plot (D) of the effect COPD of GHS(L)

## Slide 4
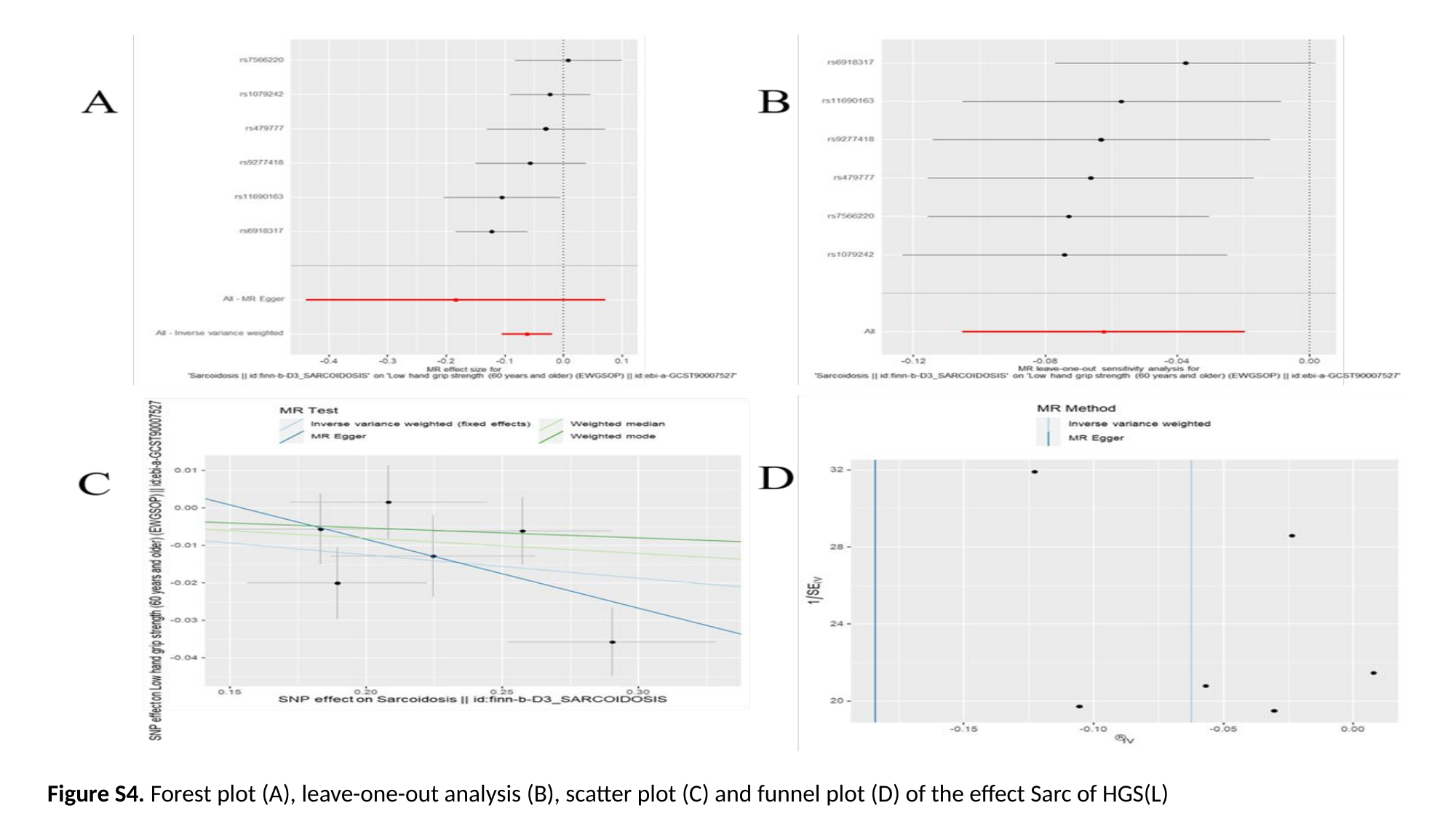

Figure S4. Forest plot (A), leave-one-out analysis (B), scatter plot (C) and funnel plot (D) of the effect Sarc of HGS(L)

## Slide 5
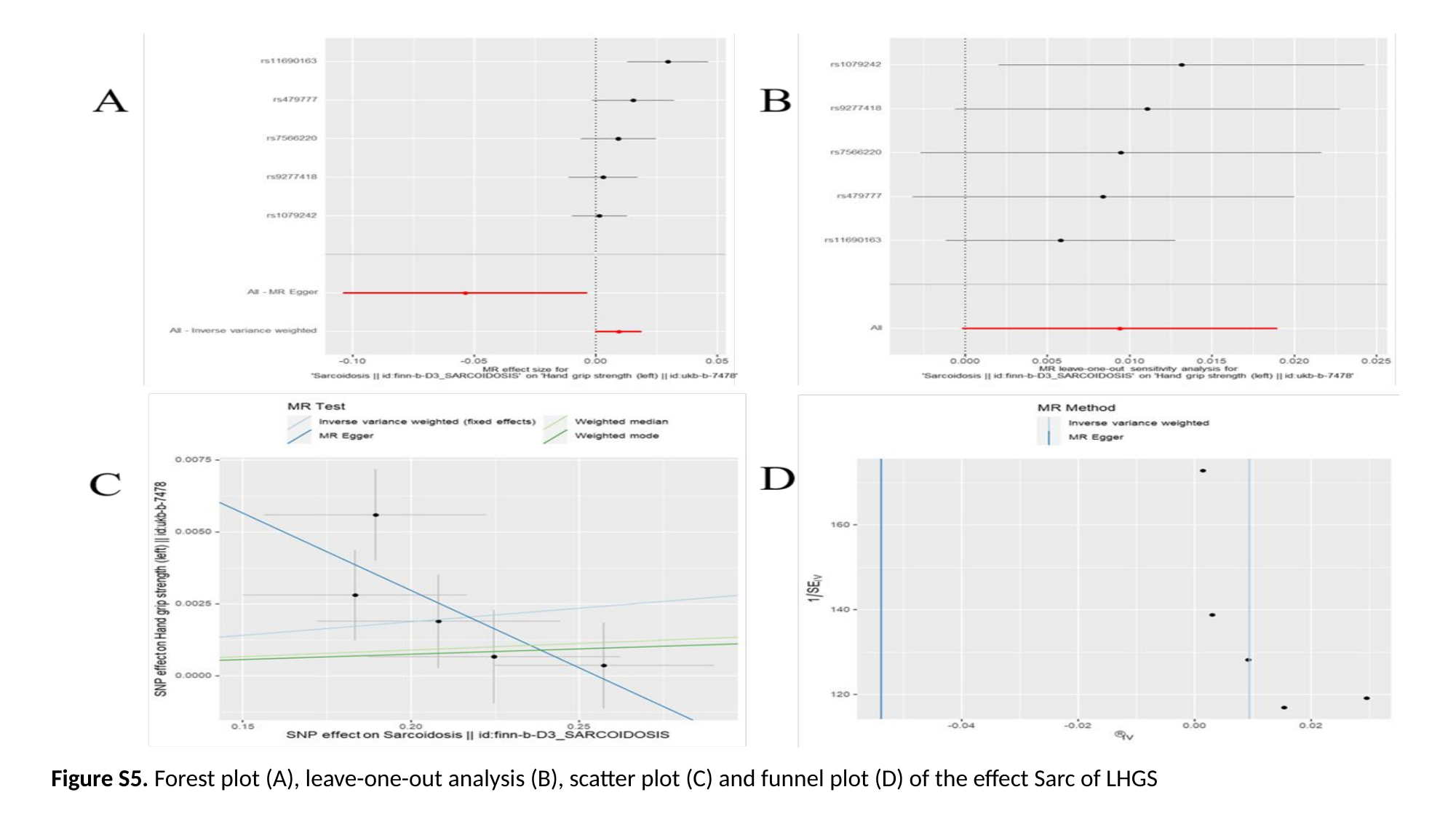

Figure S5. Forest plot (A), leave-one-out analysis (B), scatter plot (C) and funnel plot (D) of the effect Sarc of LHGS

## Slide 6
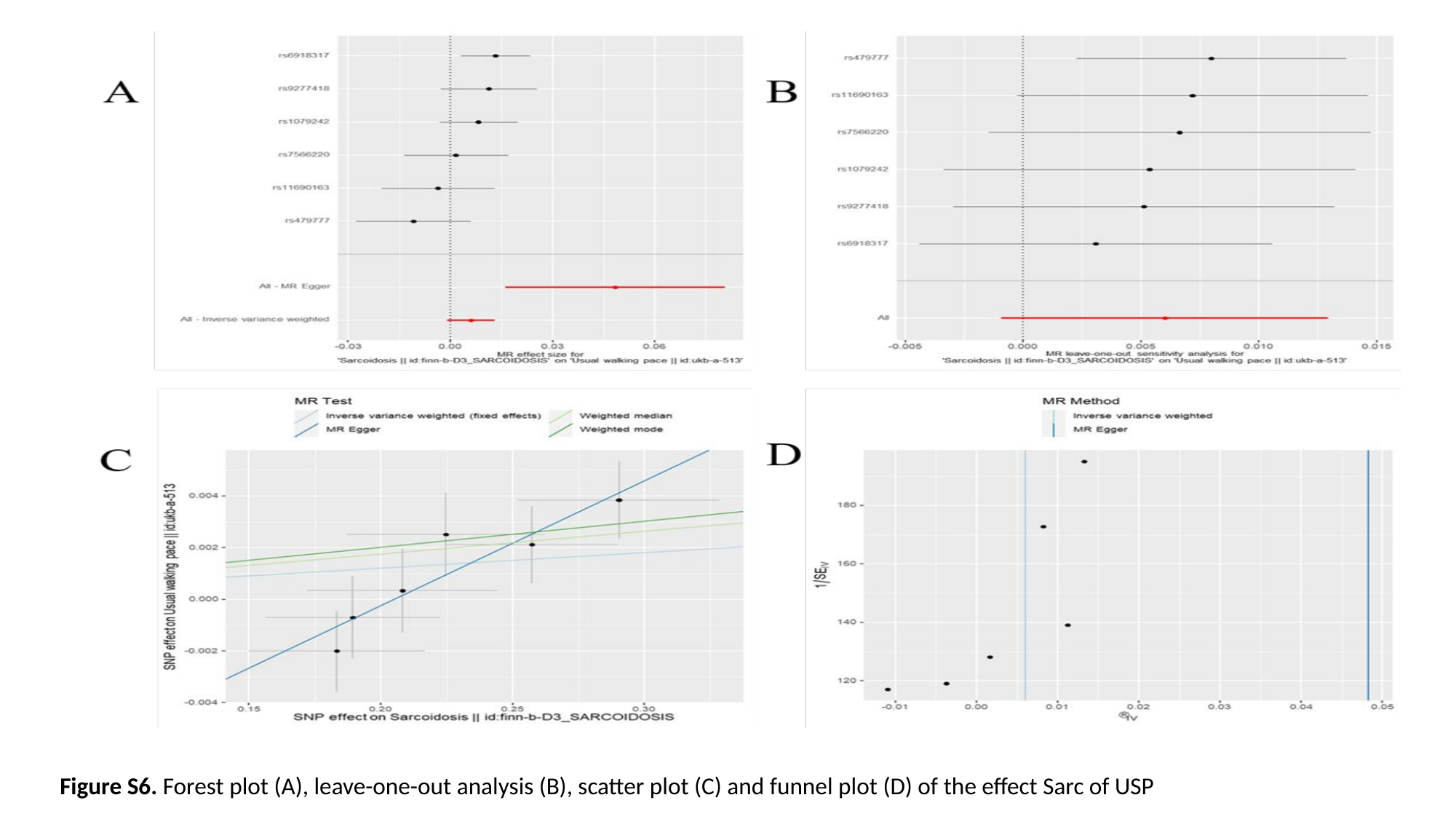

Figure S6. Forest plot (A), leave-one-out analysis (B), scatter plot (C) and funnel plot (D) of the effect Sarc of USP

## Slide 7
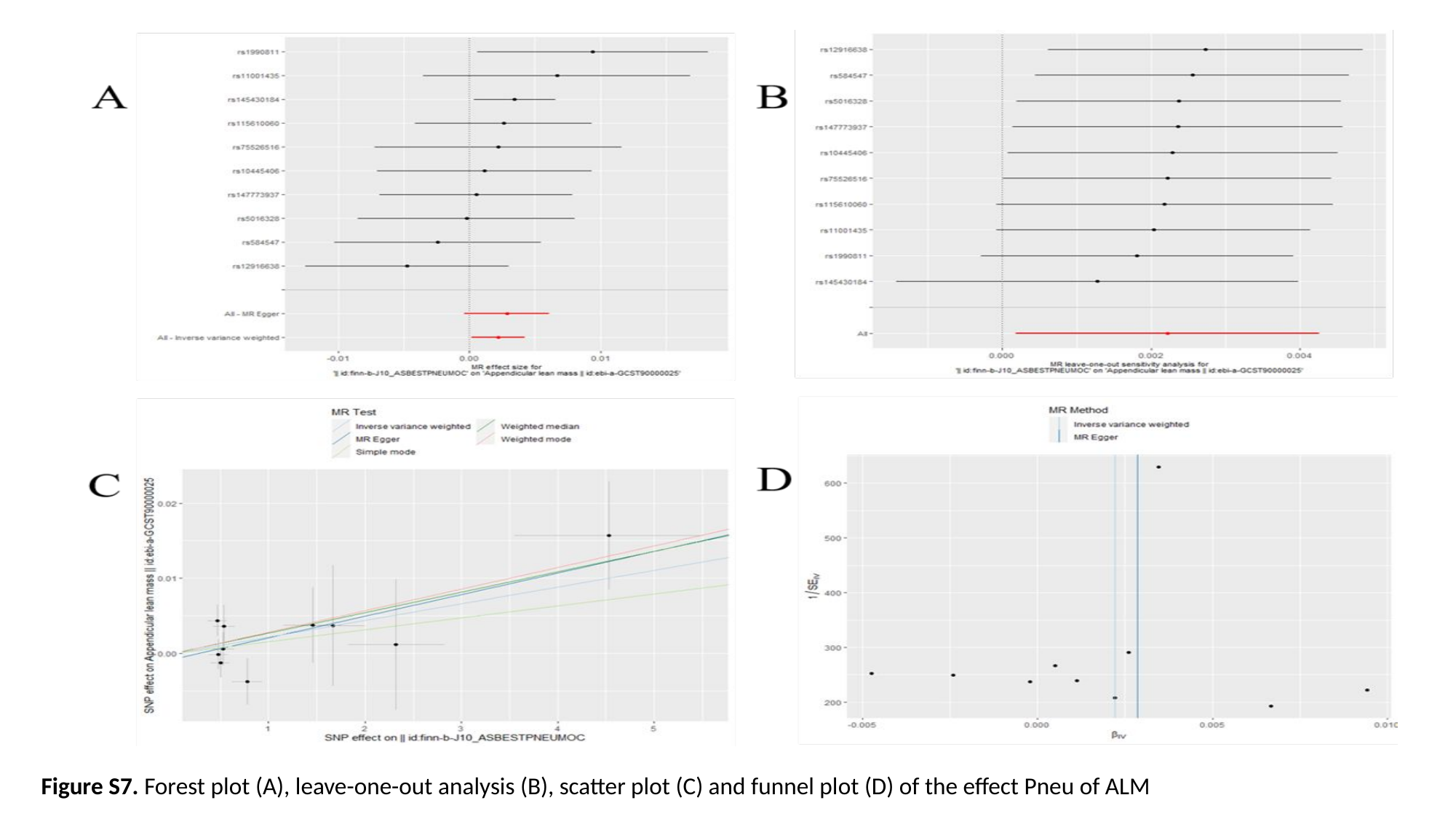

Figure S7. Forest plot (A), leave-one-out analysis (B), scatter plot (C) and funnel plot (D) of the effect Pneu of ALM

## Slide 8
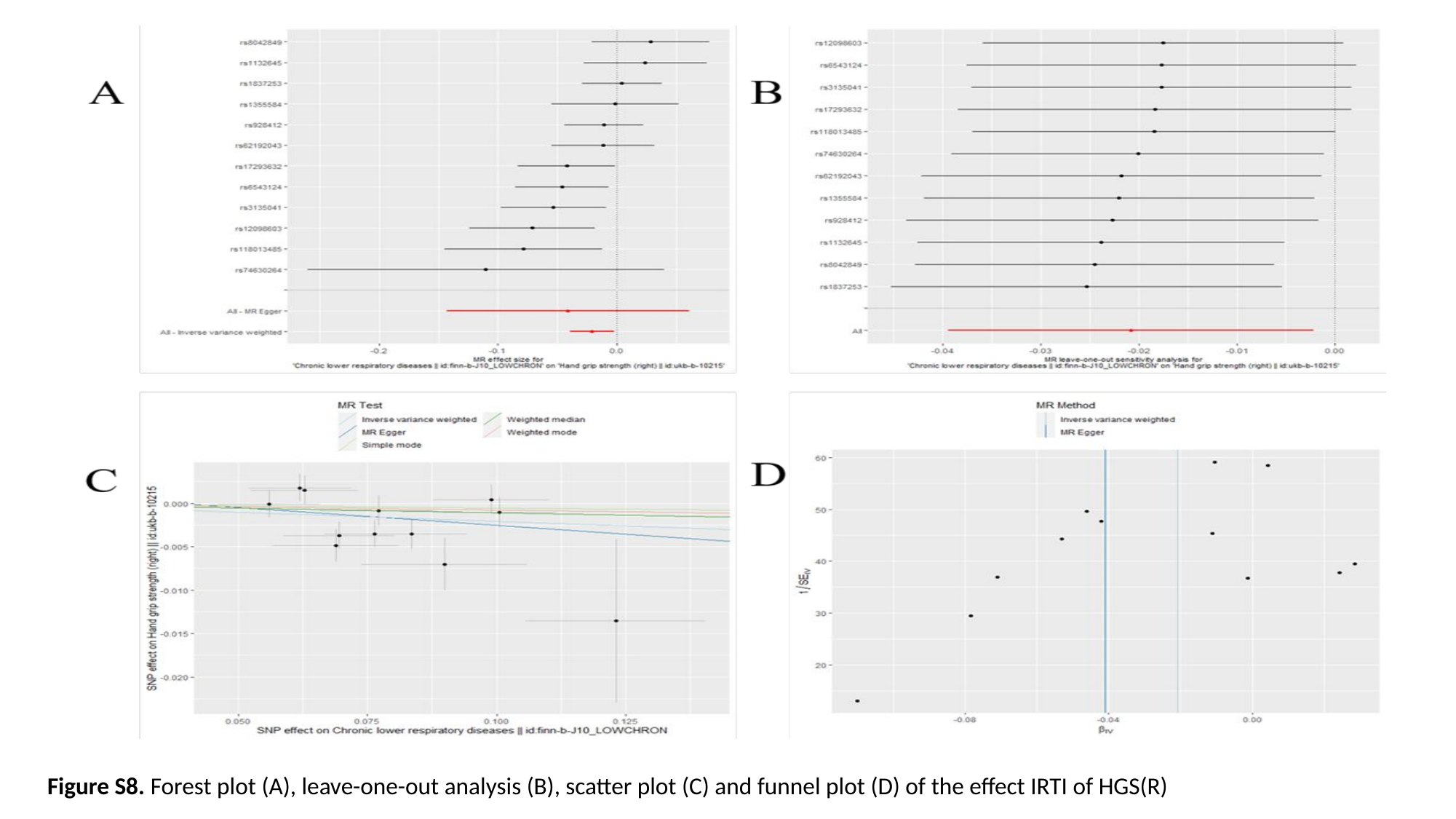

Figure S8. Forest plot (A), leave-one-out analysis (B), scatter plot (C) and funnel plot (D) of the effect IRTI of HGS(R)

## Slide 9
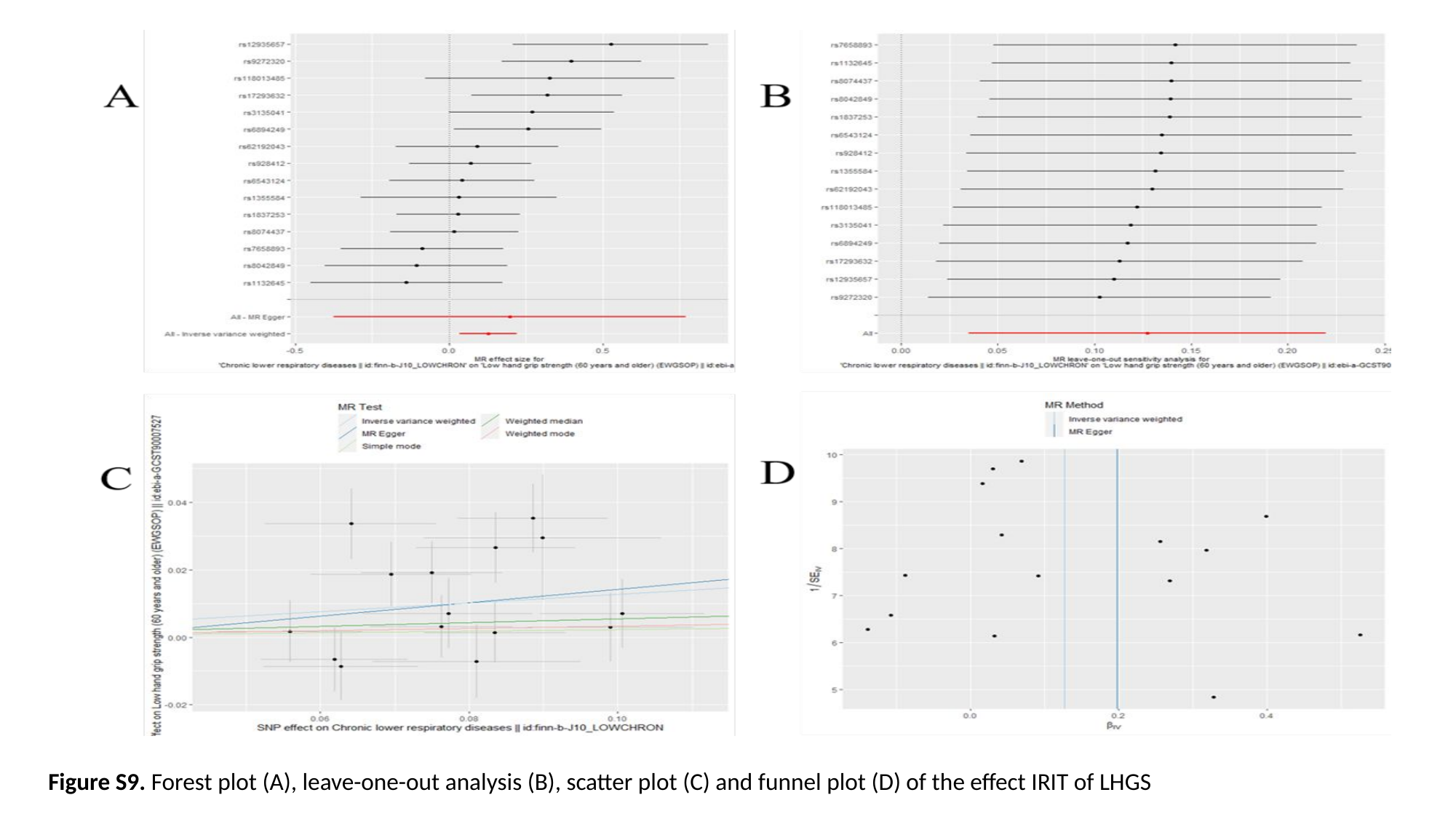

Figure S9. Forest plot (A), leave-one-out analysis (B), scatter plot (C) and funnel plot (D) of the effect IRIT of LHGS

## Slide 10
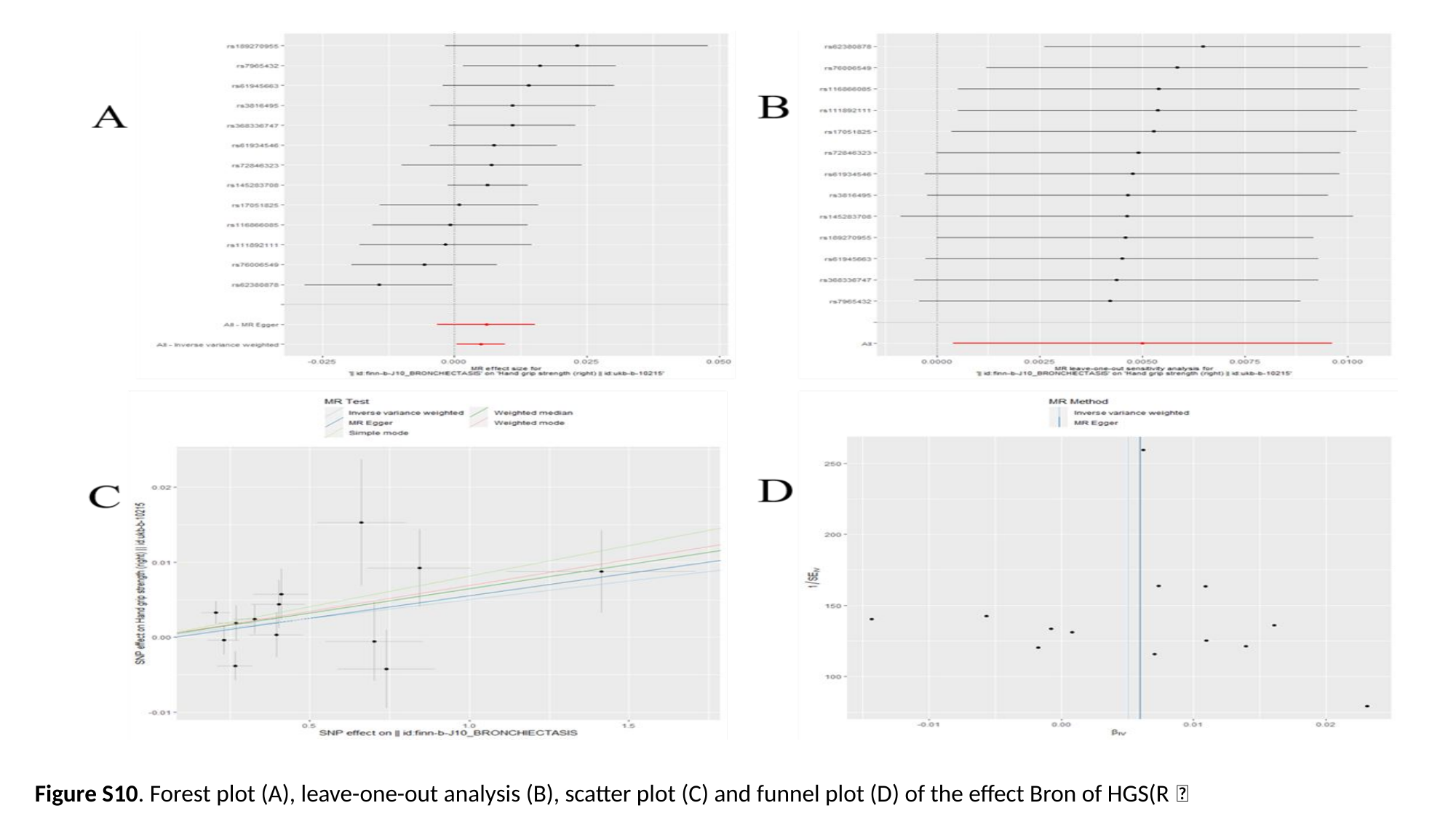

Figure S10. Forest plot (A), leave-one-out analysis (B), scatter plot (C) and funnel plot (D) of the effect Bron of HGS(R）
