# Supplementary material for: Exploring the relationship between sarcopenia and 11 respiratory diseases: a comprehensive mendelian randomization analysis
Source: Aging Clin Exp Res. 2024 Oct 12;36(1):205. doi: 10.1007/s40520-024-02855-y (PMC11470909; doi:10.1007/s40520-024-02855-y)
Supplement: Supplementary file 2 — Supplementary file2 (PPTX 4385 KB) [file 40520_2024_2855_MOESM2_ESM.pptx]

## Slide 1
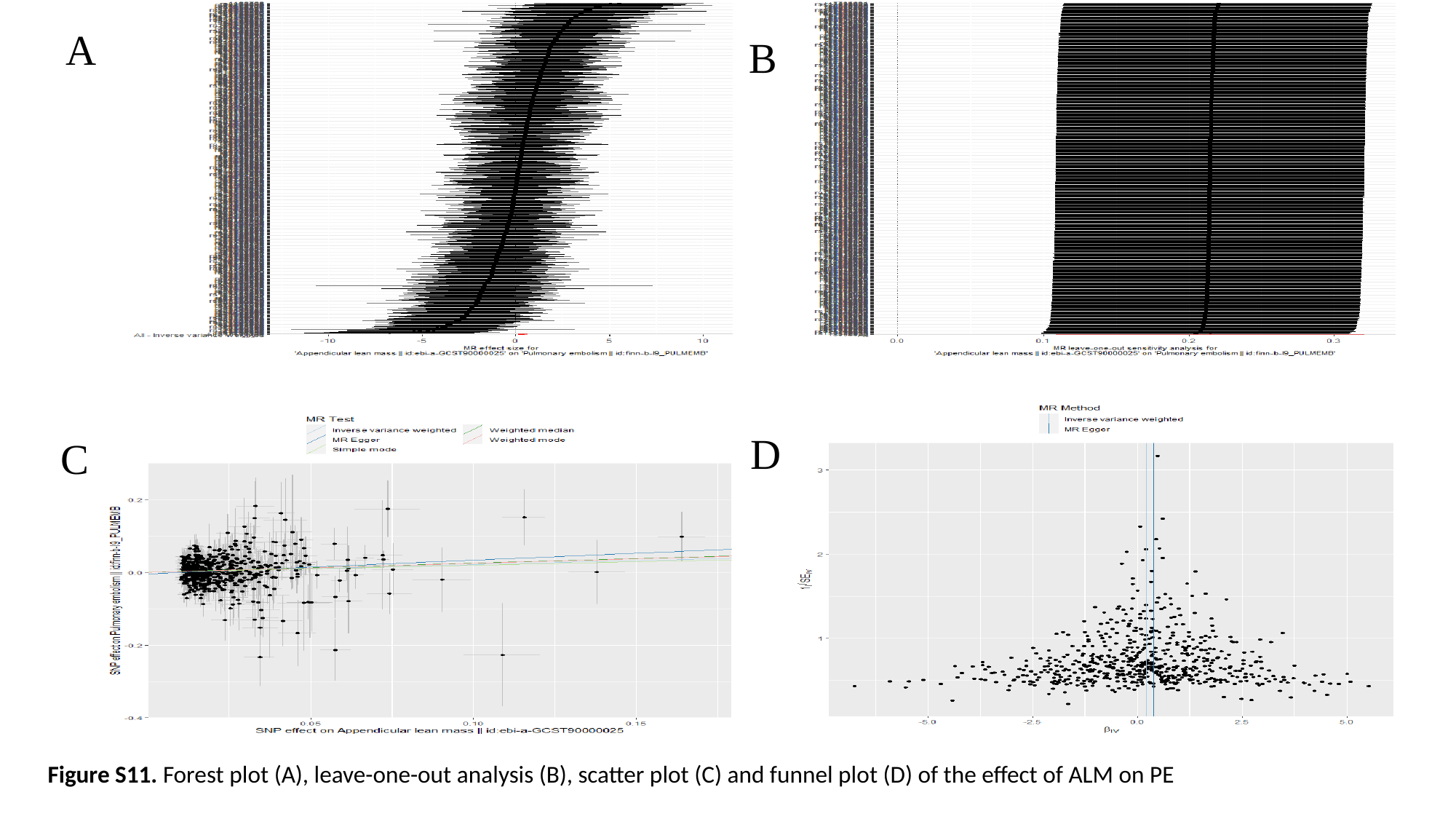

A
B
D
C
Figure S11. Forest plot (A), leave-one-out analysis (B), scatter plot (C) and funnel plot (D) of the effect of ALM on PE

## Slide 2
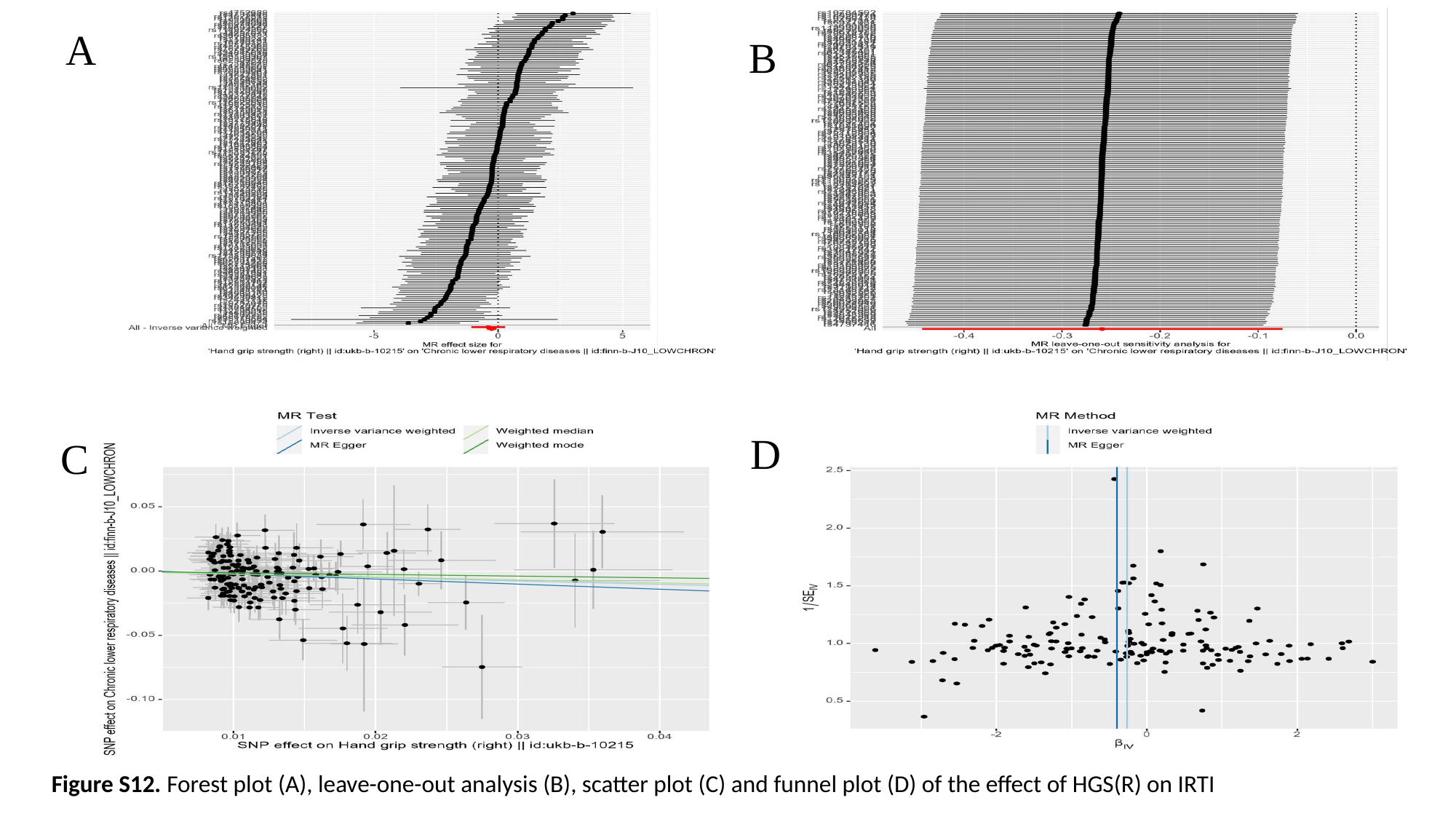

A
B
D
C
Figure S12. Forest plot (A), leave-one-out analysis (B), scatter plot (C) and funnel plot (D) of the effect of HGS(R) on IRTI

## Slide 3
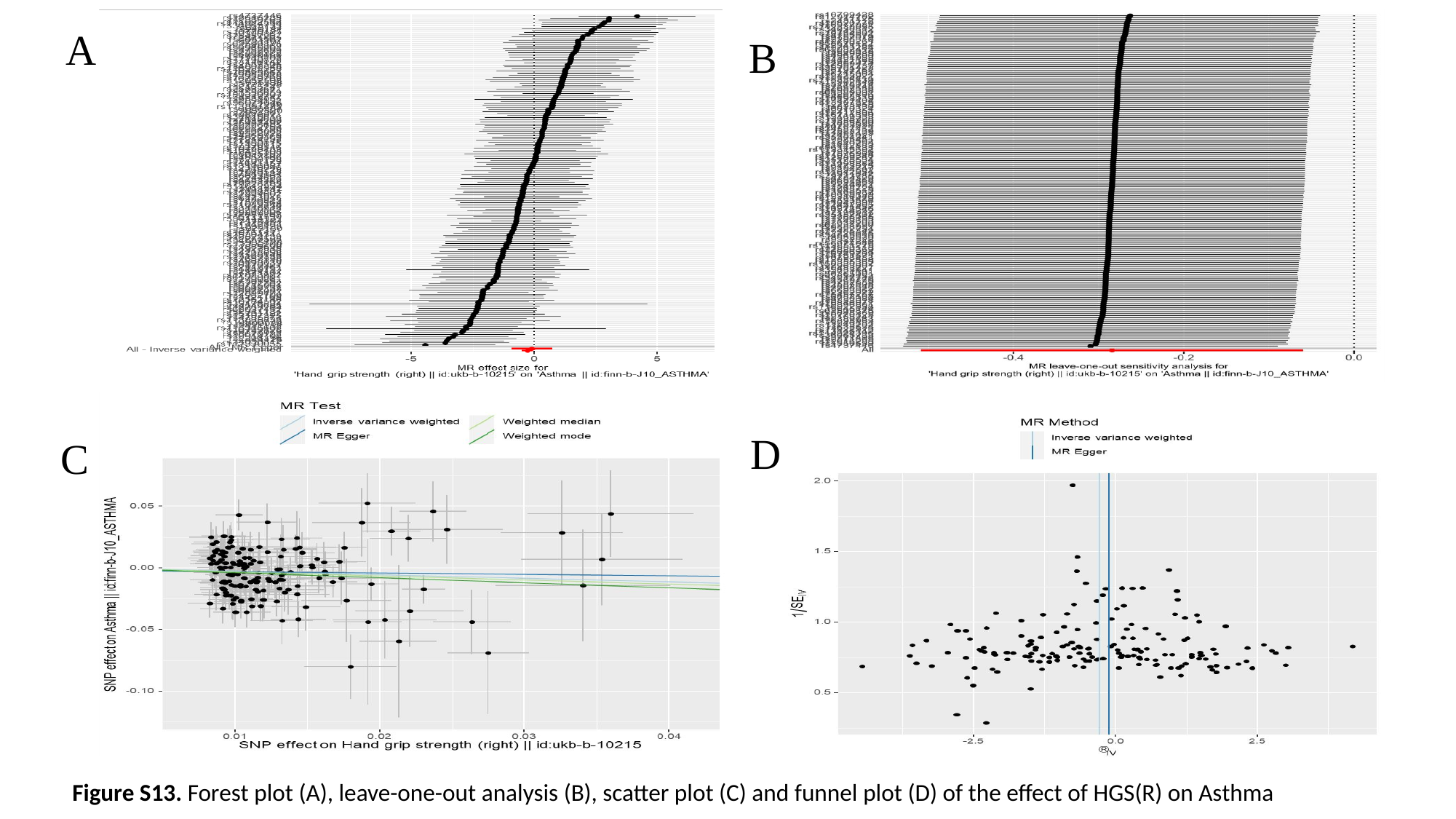

A
B
D
C
Figure S13. Forest plot (A), leave-one-out analysis (B), scatter plot (C) and funnel plot (D) of the effect of HGS(R) on Asthma

## Slide 4
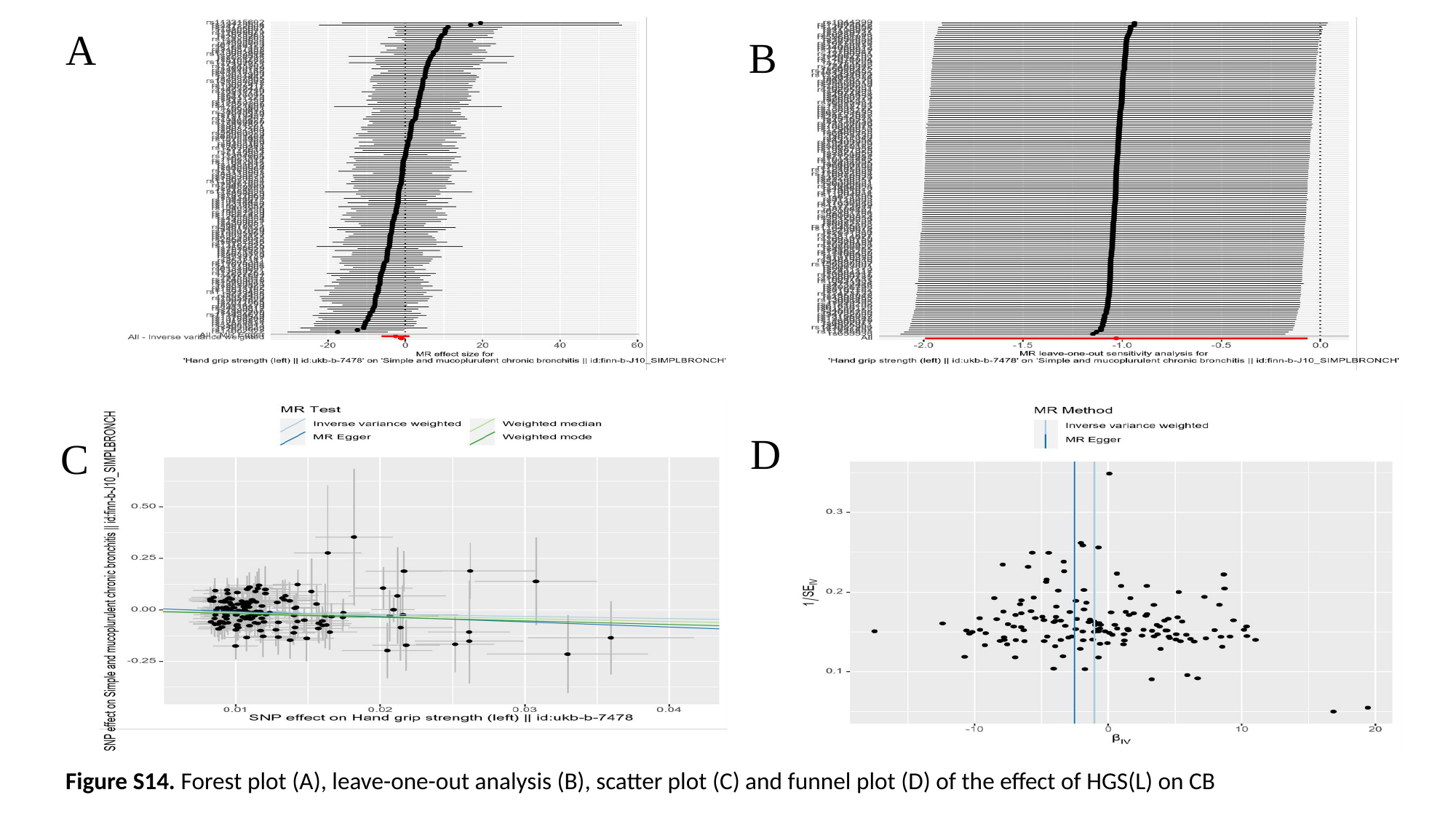

A
B
D
C
Figure S14. Forest plot (A), leave-one-out analysis (B), scatter plot (C) and funnel plot (D) of the effect of HGS(L) on CB

## Slide 5
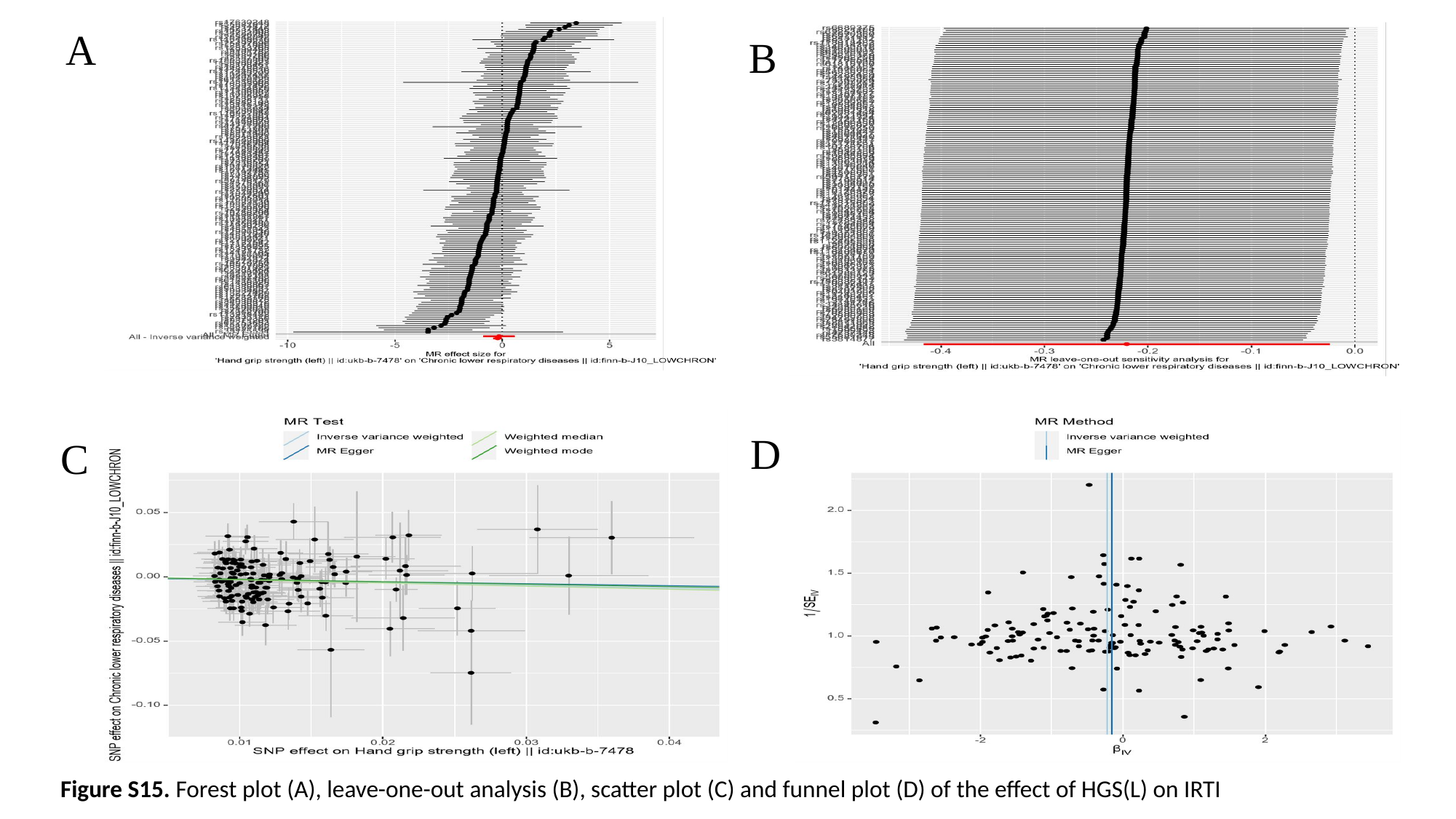

A
B
D
C
Figure S15. Forest plot (A), leave-one-out analysis (B), scatter plot (C) and funnel plot (D) of the effect of HGS(L) on IRTI

## Slide 6
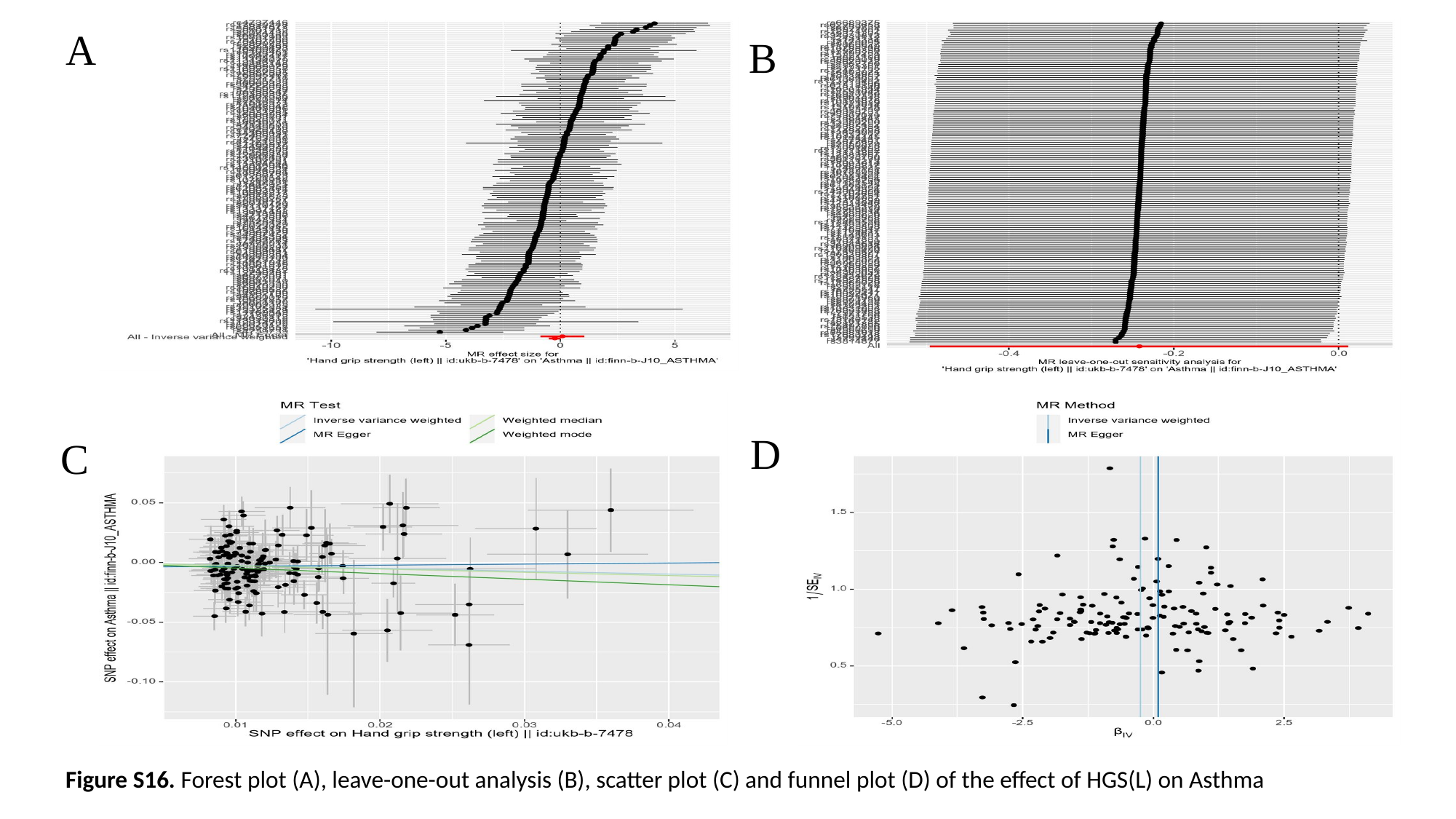

A
B
D
C
Figure S16. Forest plot (A), leave-one-out analysis (B), scatter plot (C) and funnel plot (D) of the effect of HGS(L) on Asthma

## Slide 7
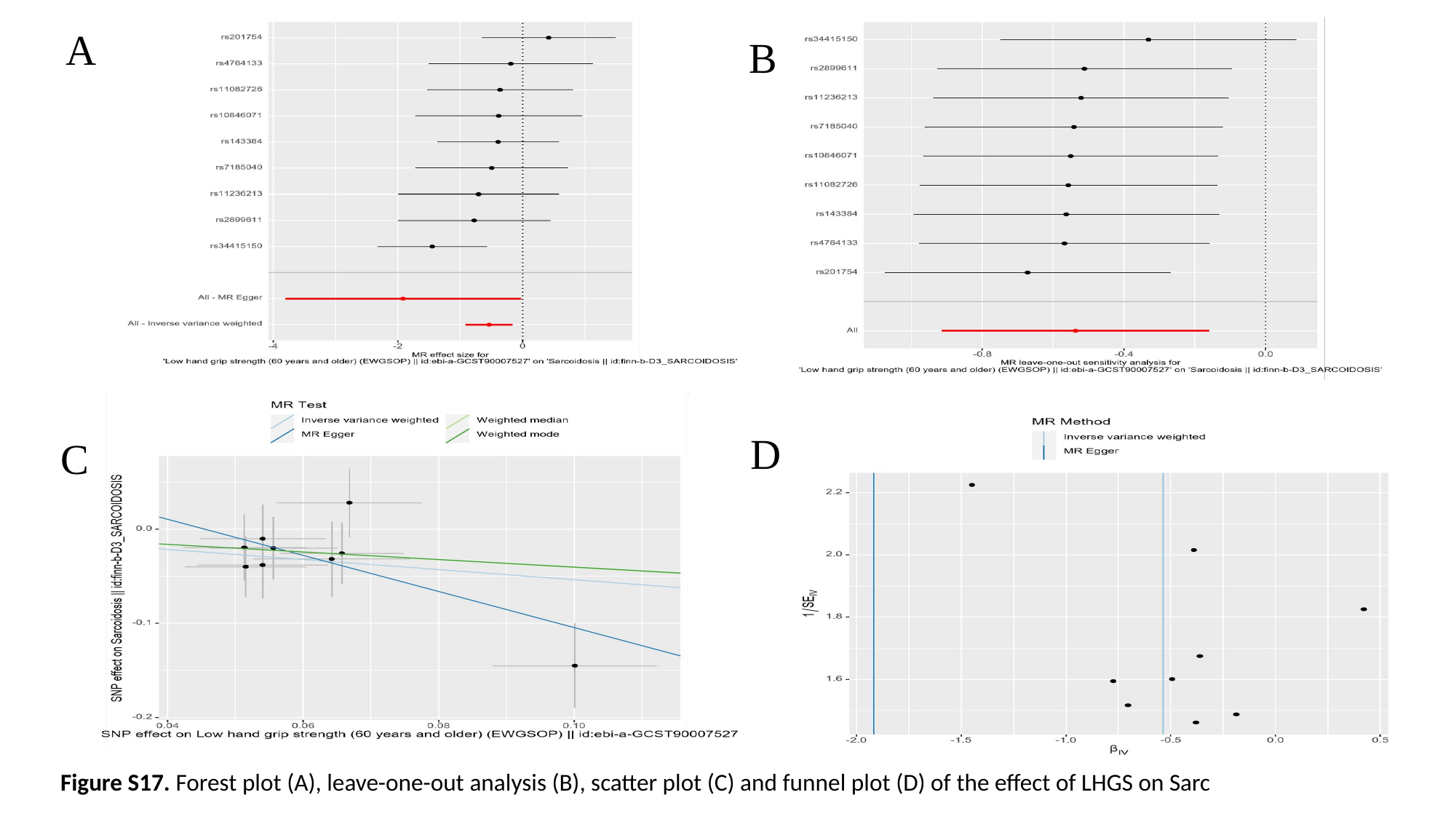

A
B
D
C
Figure S17. Forest plot (A), leave-one-out analysis (B), scatter plot (C) and funnel plot (D) of the effect of LHGS on Sarc

## Slide 8
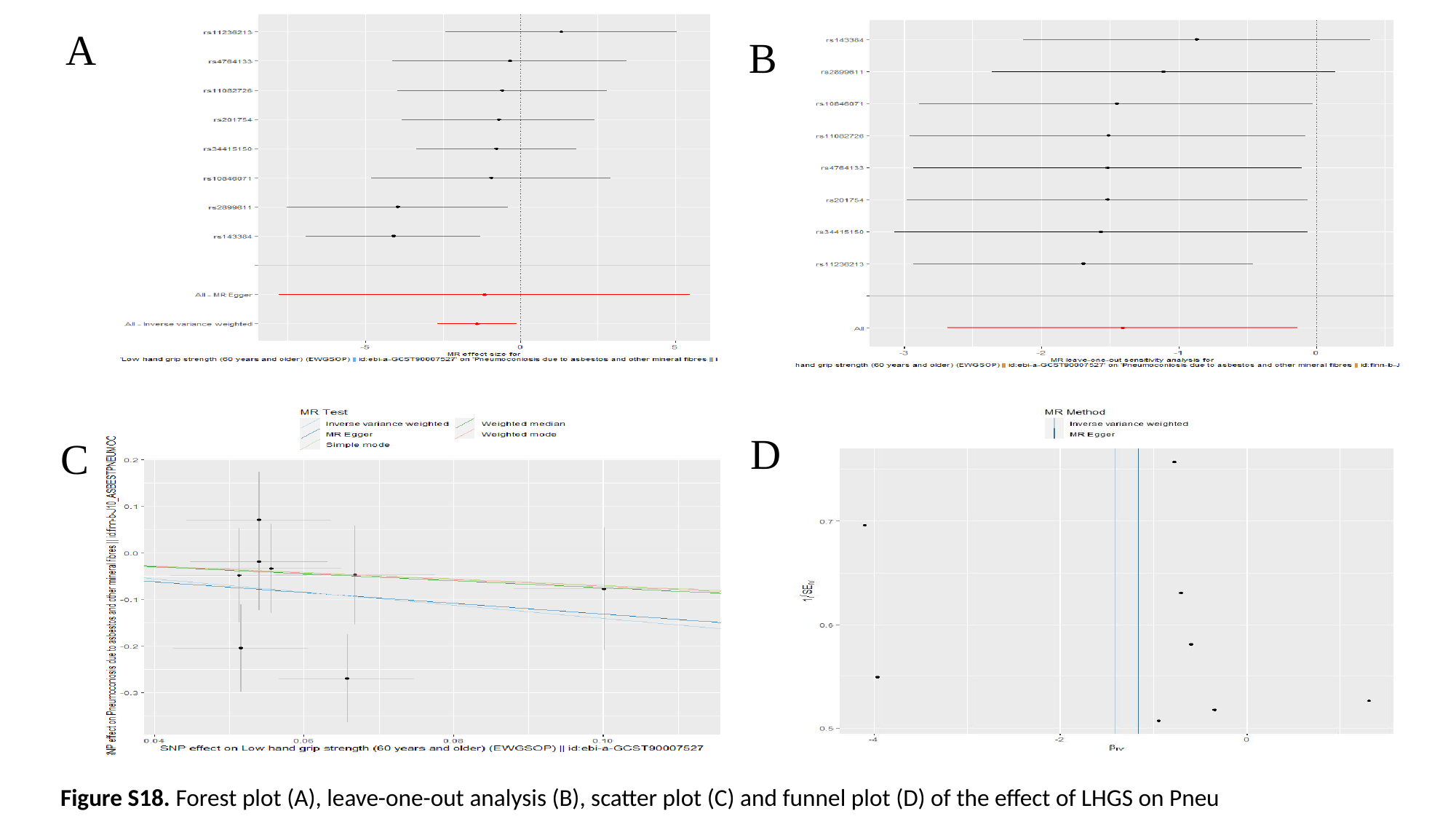

A
B
D
C
Figure S18. Forest plot (A), leave-one-out analysis (B), scatter plot (C) and funnel plot (D) of the effect of LHGS on Pneu

## Slide 9
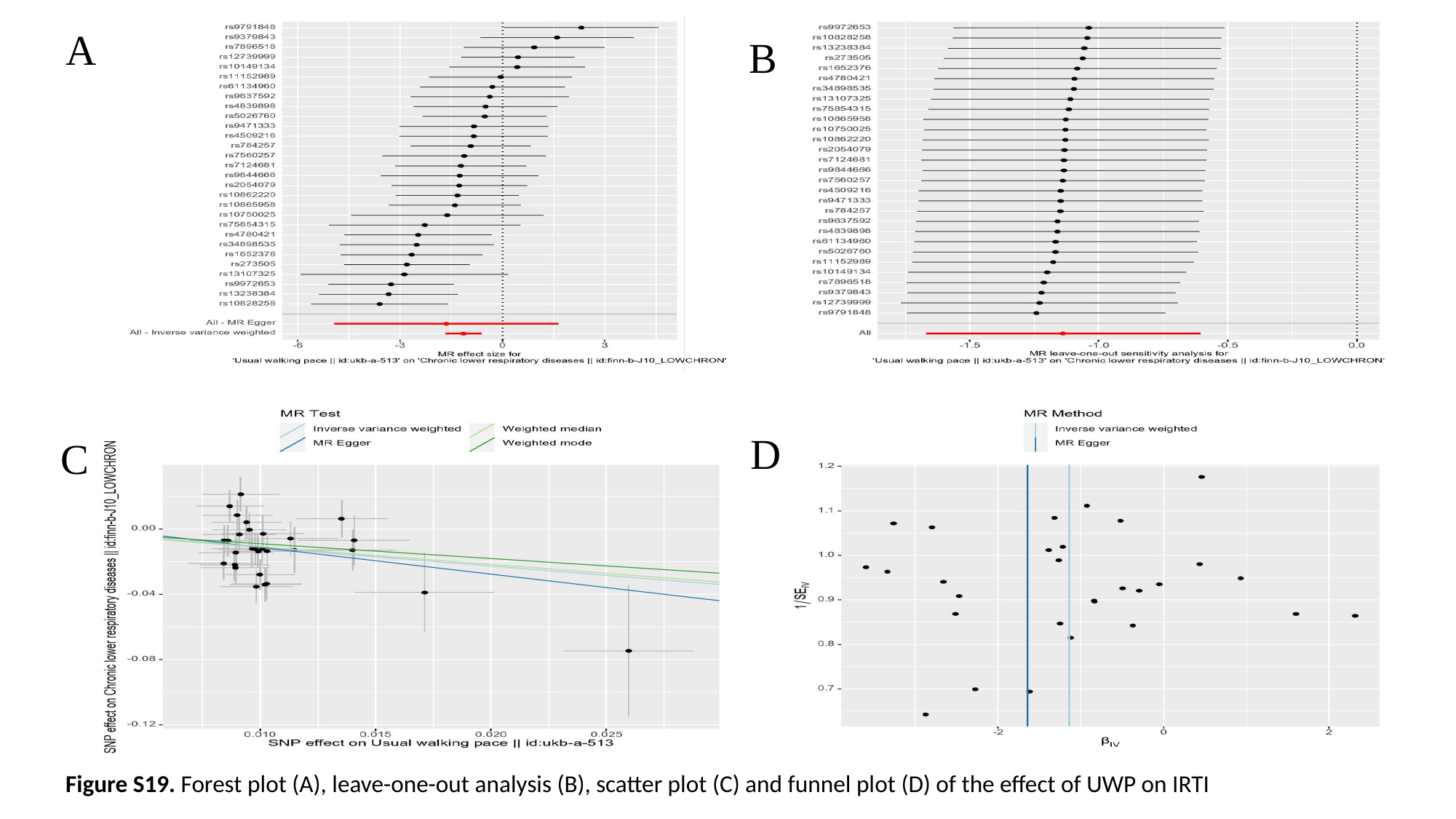

A
B
D
C
Figure S19. Forest plot (A), leave-one-out analysis (B), scatter plot (C) and funnel plot (D) of the effect of UWP on IRTI

## Slide 10
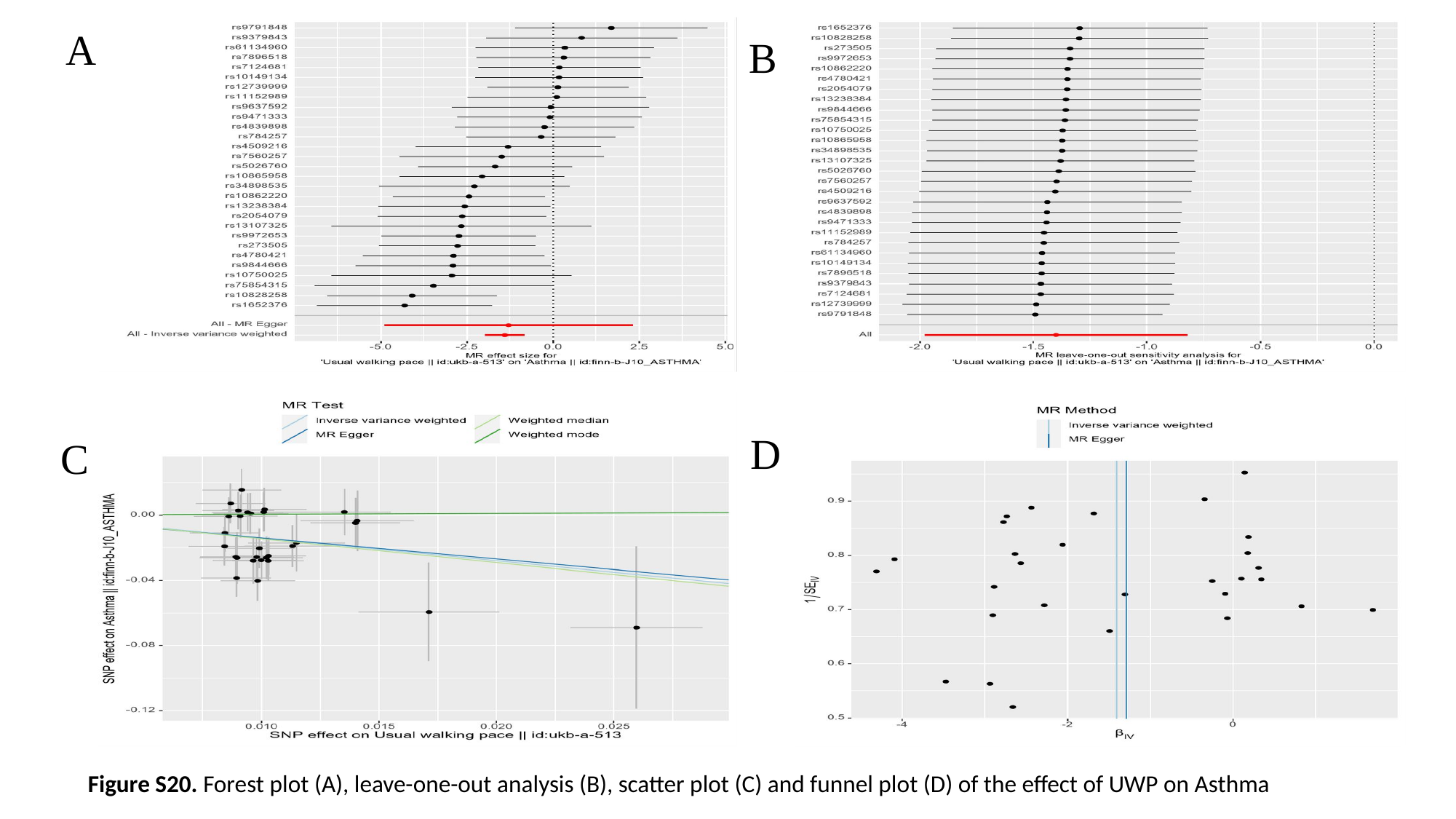

A
B
D
C
Figure S20. Forest plot (A), leave-one-out analysis (B), scatter plot (C) and funnel plot (D) of the effect of UWP on Asthma

## Slide 11
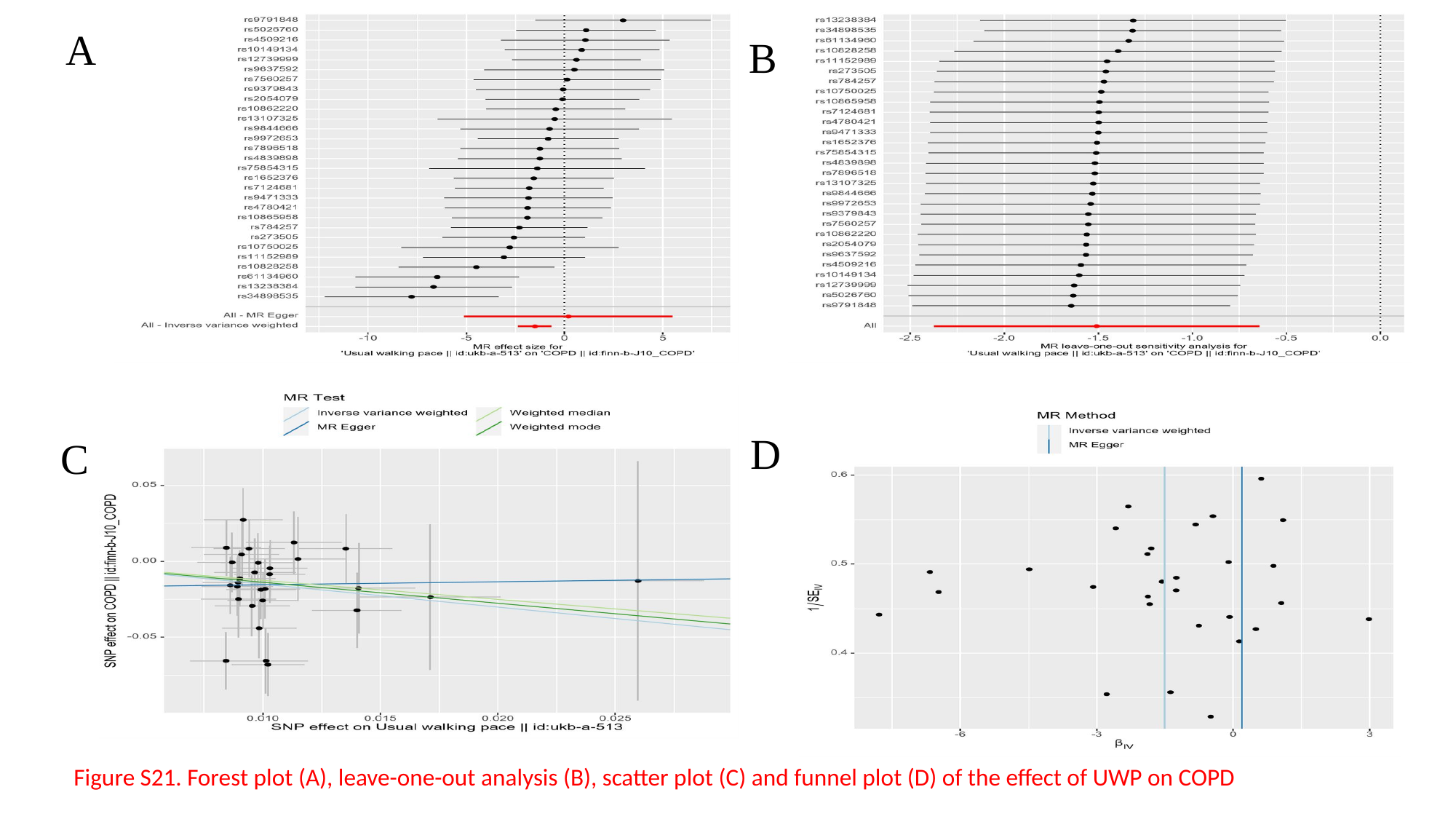

A
B
D
C
Figure S21. Forest plot (A), leave-one-out analysis (B), scatter plot (C) and funnel plot (D) of the effect of UWP on COPD

## Slide 12
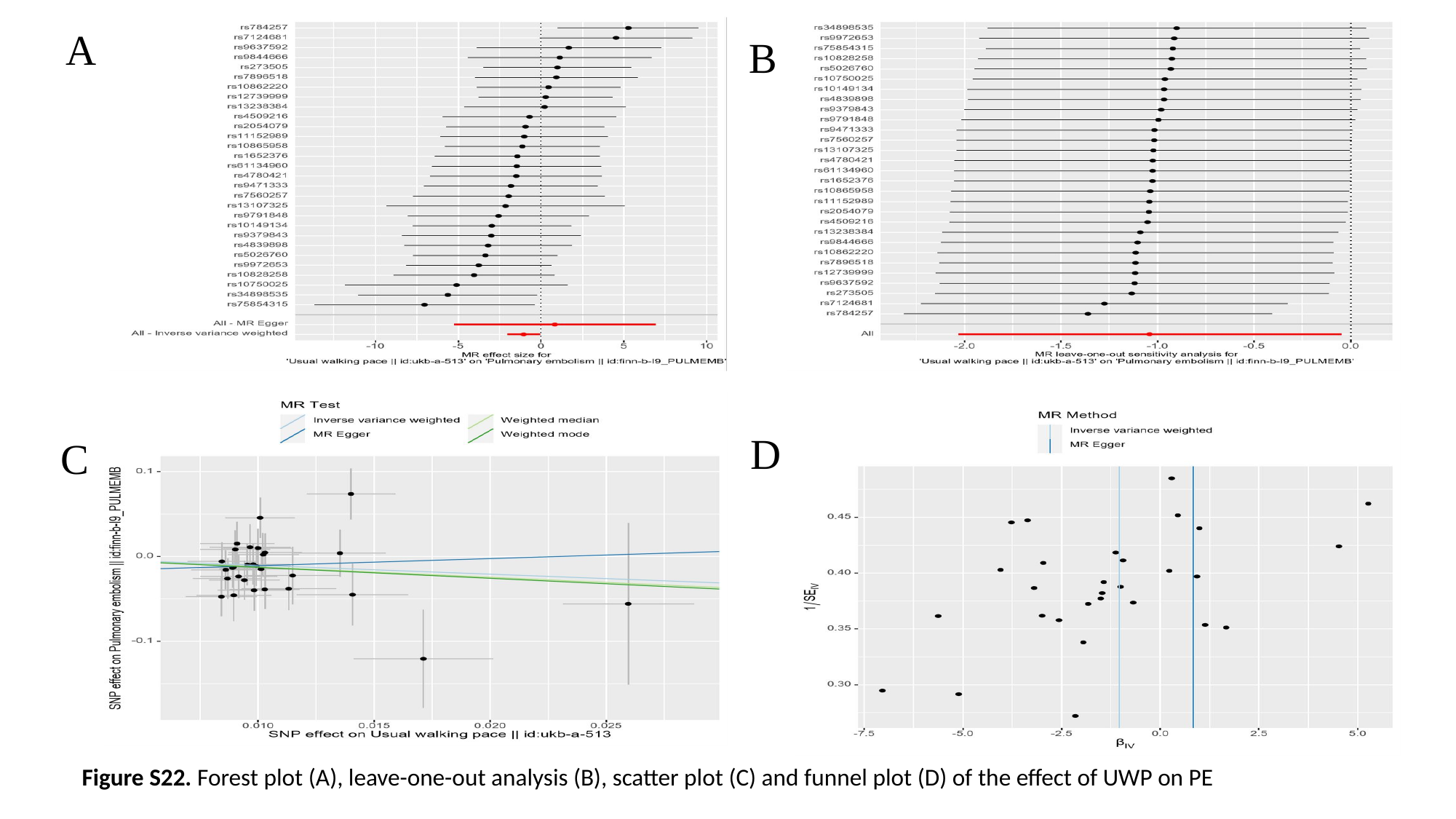

A
B
D
C
Figure S22. Forest plot (A), leave-one-out analysis (B), scatter plot (C) and funnel plot (D) of the effect of UWP on PE
